# Crystal structures and Hirshfeld surface analyses of methyl 4-{2,2-di­chloro-1-[(*E*)-phenyl­diazen­yl]eth­enyl}benzoate, methyl 4-{2,2-di­chloro-1-[(*E*)-(4-methyl­phen­yl)diazen­yl]ethen­yl}benzoate and methyl 4-{2,2-di­chloro-1-[(*E*)-(3,4-di­methyl­phen­yl)diazen­yl]ethen­yl}benzoate

**DOI:** 10.1107/S2056989024000732

**Published:** 2024-01-26

**Authors:** Namiq Q. Shikhaliyev, Shafiga A. İbrahimova, Gulnar T. Atakishiyeva, Nigar E. Ahmedova, Gulnara V. Babayeva, Victor N. Khrustalev, Zeliha Atioğlu, Mehmet Akkurt, Ajaya Bhattarai

**Affiliations:** a Organic Chemistry Department, Baku State University, Z. Khalilov str. 23, AZ 1148 Baku, Azerbaijan; b Department of Analytical and Organic Chemistry, Azerbaijan State Pedagogical University, Uzeyir Hajibeyli str., 68, Baku, Azerbaijan; c Peoples’ Friendship University of Russia (RUDN University), Miklukho-Maklay St. 6, Moscow, 117198, Russian Federation; d N. D. Zelinsky Institute of Organic Chemistry RAS, Leninsky Prosp. 47, Moscow, 119991, Russian Federation; e Department of Aircraft Electrics and Electronics, School of Applied Sciences, Cappadocia University, Mustafapaşa, 50420 Ürgüp, Nevşehir, Türkiye; f Department of Physics, Faculty of Sciences, Erciyes University, 38039 Kayseri, Türkiye; g Department of Chemistry, M.M.A.M.C (Tribhuvan University) Biratnagar, Nepal; Vienna University of Technology, Austria

**Keywords:** crystal structure, C—H⋯N hydrogen bonds, C—H⋯π inter­actions, C—Cl⋯π inter­actions, Hirshfeld surface analysis

## Abstract

The crystal structures and Hirshfeld surface analyses of three similar azo compounds are reported. Methyl 4-{2,2-di­chloro-1-[(*E*)-phenyl­diazen­yl]ethen­yl}benzoate, C_16_H_12_Cl_2_N_2_O_2_, (**I**), and methyl 4-{2,2-di­chloro-1-[(*E*)-(4-methyl­phen­yl)diazen­yl]ethen­yl}benzoate, C_17_H_14_Cl_2_N_2_O_2_, (**II**), crystallize in the space group *P*2_1_/*c* with *Z* = 4, and methyl 4-{2,2-di­chloro-1-[(*E*)-(3,4-di­methyl­phen­yl)diazen­yl]ethen­yl}benzoate, C_18_H_16_Cl_2_N_2_O_2_, (**III**), in the space group *P*  with *Z* = 2. In the crystal of (**I**), mol­ecules are linked by C—H⋯N hydrogen bonds, forming chains with *C*(6) motifs parallel to the *b* axis. Short inter­molecular Cl⋯O contacts of 2.8421 (16) Å and weak van der Waals inter­actions between these chains stabilize the crystal structure. In (**II**), mol­ecules are linked by C—H⋯O hydrogen bonds and C—Cl⋯π inter­actions, forming layers parallel to (010). Weak van der Waals inter­actions between these layers consolidate the mol­ecular packing. In (**III**), mol­ecules are linked by C—H⋯π and C—Cl⋯π inter­actions forming chains parallel to [011]. Furthermore, these chains are connected by C—Cl⋯π inter­actions parallel to the *a* axis, forming (0  1) layers. The stability of the mol­ecular packing is ensured by van der Waals forces between these layers.

## Abstract 2

In the crystal of methyl 4-{2,2-di­chloro-1-[(*E*)-phenyl­diazen­yl]ethen­yl}benzoate, mol­ecules are linked by C—H⋯N hydrogen bonds, forming chains with *C*(6) motifs parallel to the *b* axis. In methyl 4-{2,2-di­chloro-1-[(*E*)-(4-methyl­phen­yl)diazen­yl]ethen­yl}benzoate, mol­ecules are linked by C—H⋯O hydrogen bonds and C—Cl⋯π inter­actions, forming layers parallel to (010). In methyl 4-{2,2-di­chloro-1-[(*E*)-(3,4-di­methyl­phen­yl)diazen­yl]ethen­yl}benzoate, mol­ecules are linked by C—H⋯π and C—Cl⋯π inter­actions, forming chains parallel to [011].

## 1. Chemical context

**Figure 1:**
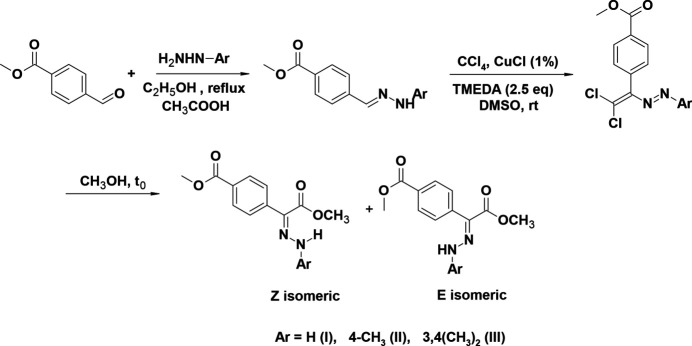
Reaction scheme for synthesis of azo dyes with the methyl­benzoate fragment.

When manufacturing new insecticides and pesticides, it is important that they are harmless to the environment and humans. This condition is fulfilled for most biopesticides. For example, methyl­benzoate is considered to be a bio-insecticide (Mostafiz *et al.*, 2022 ▸; Chen *et al.*, 2015 ▸; Damalas & Eleftherohorinos, 2011 ▸; Goulson, 2013 ▸; Naqqash *et al.*, 2016 ▸; Zikankuba *et al.*, 2019 ▸) and is reported to be less harmful to the human body and the environment. Methyl­benzoate is also found as a metabolite in plants and has an attractive odour to insects. At the same time, methyl benzoate is very effective as a pesticide against agricultural and warehouse pests (Isman, 2015 ▸, 2020 ▸; Pavela, 2016 ▸; Pavela & Benelli, 2016 ▸). It can therefore be concluded that methyl benzoate and its derivatives might exhibit applications as pesticides and insecticides, and the synthesis of such or related biopesticides is an urgent issue. Taking this into account, we focused on phenyl­hydrazones that were obtained from the reaction of methyl 4-formyl­benzoate with the corresponding phenyl­hydrazines (Maharramov *et al.*, 2018 ▸; Nenajdenko *et al.*, 2020 ▸, Shikhaliyev *et al.*, 2018 ▸, 2019*a* ▸,*b* ▸, 2021*a* ▸,*b* ▸,*c* ▸), and the synthesis of methyl (*E*)-4-{2,2-di­chloro-1-[(substitutedphen­yl)diazen­yl]vin­yl}benzoate derivatives was carried out from the reaction of the latter with CCl_4_. The here synthesized di­chlorodi­aza­diene derivatives (**I**), (**II**) and (**III**) and aryl­hydrazo derivatives of α-keto esters obtained from their solvolysis are intended to be studied in future research as compounds with the above-mentioned properties (Fig. 1 ▸). 
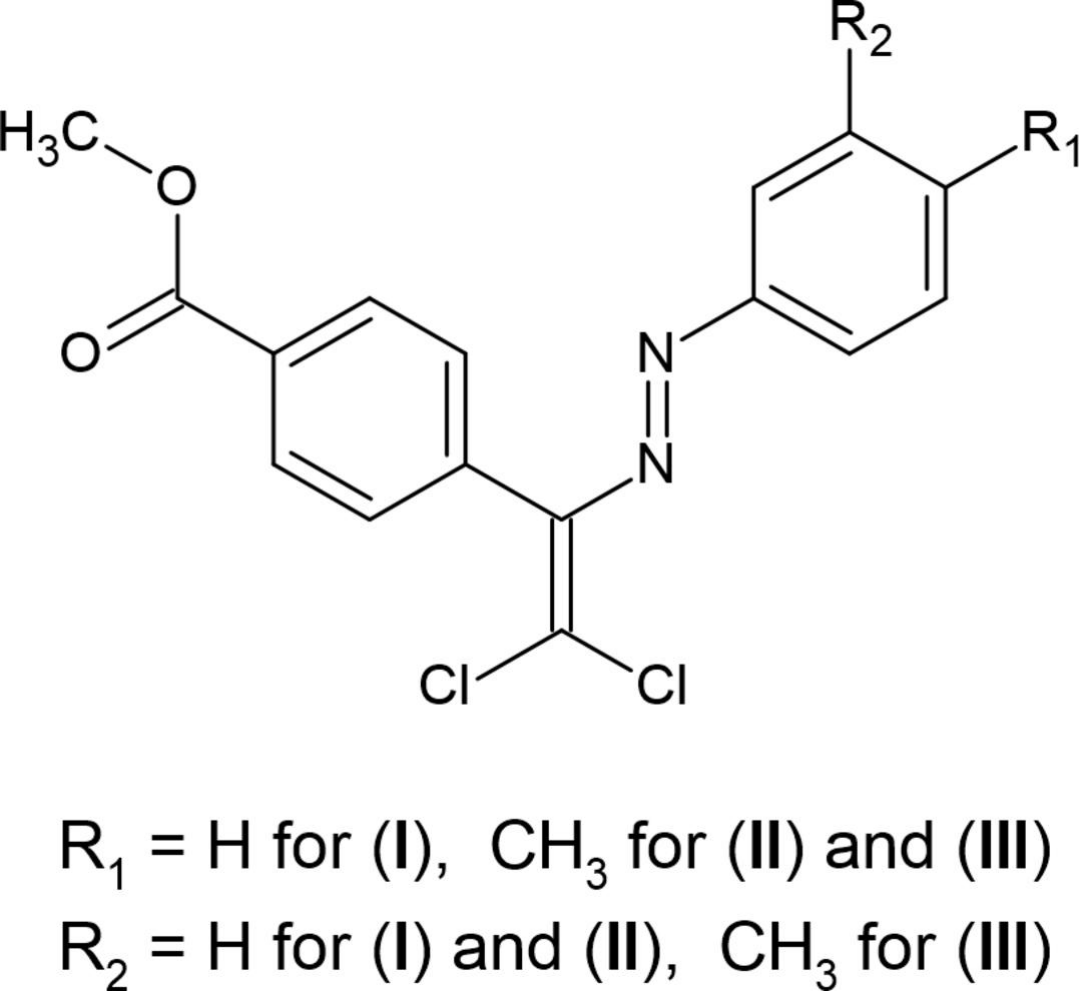


## 2. Structural commentary

**Figure 2:**
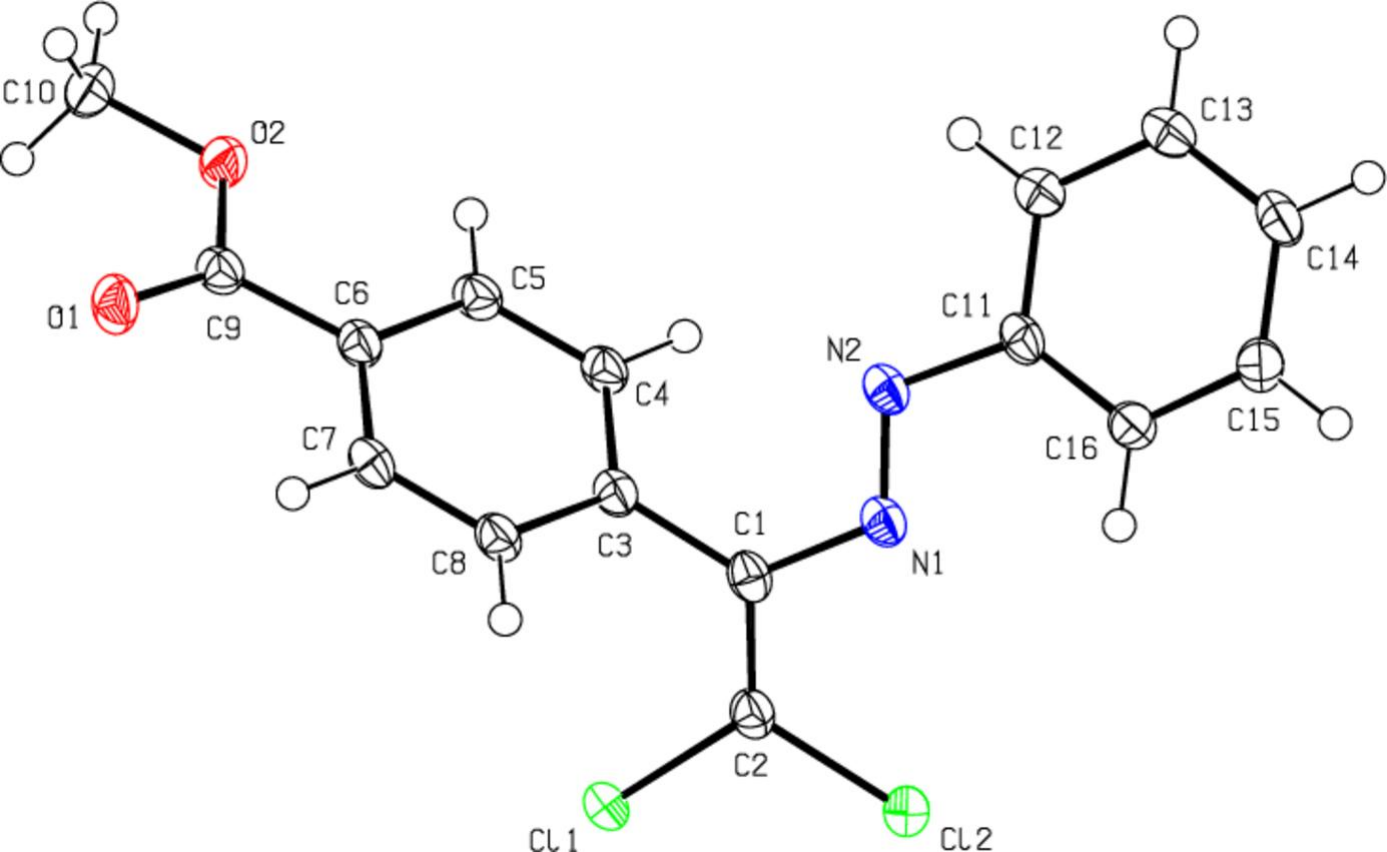
The mol­ecular structure of (**I**), showing the atom labelling and displacement ellipsoids drawn at the 50% probability level.

**Figure 3:**
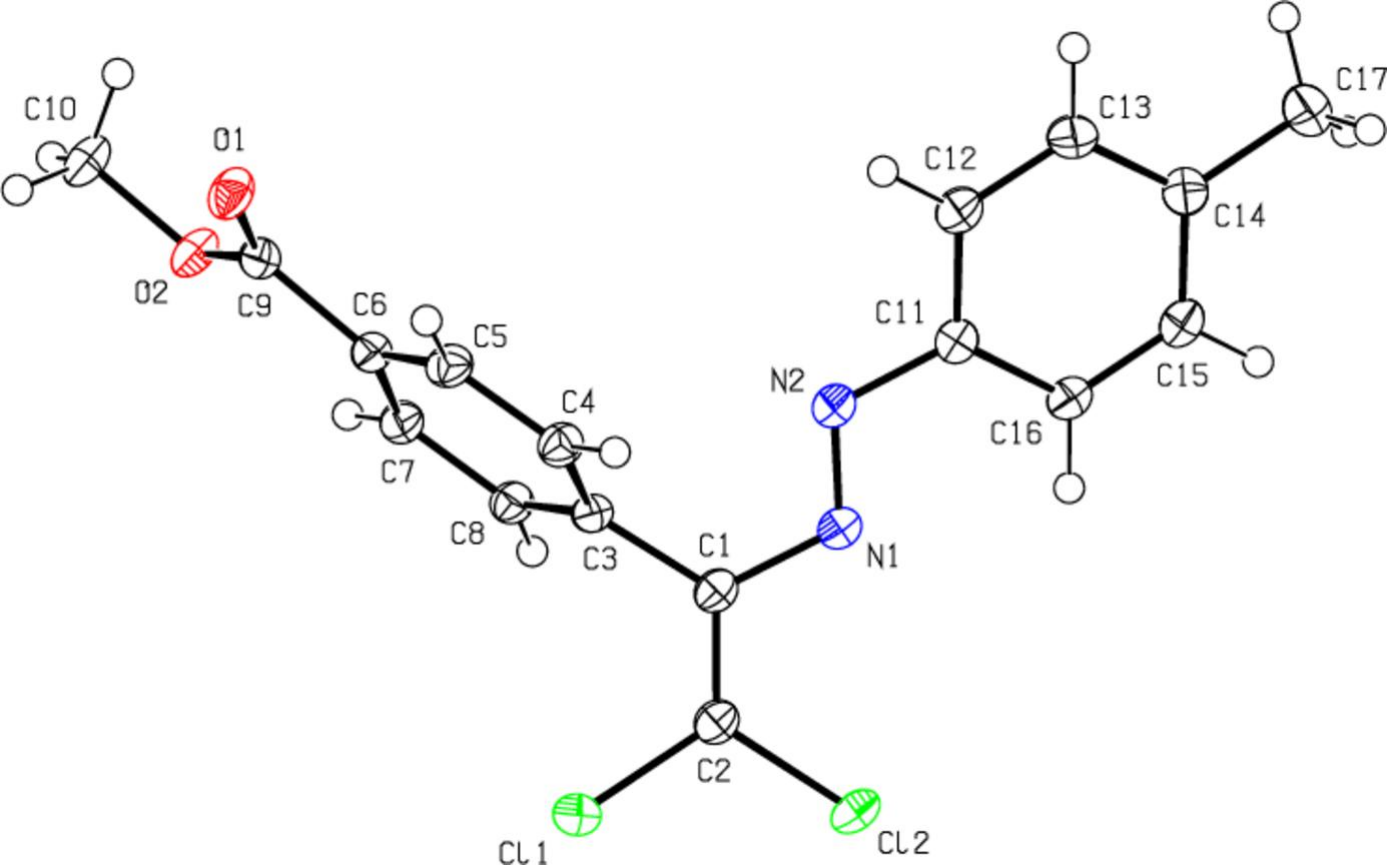
The mol­ecular structure of (**II**), showing the atom labelling and displacement ellipsoids drawn at the 50% probability level.

**Figure 4:**
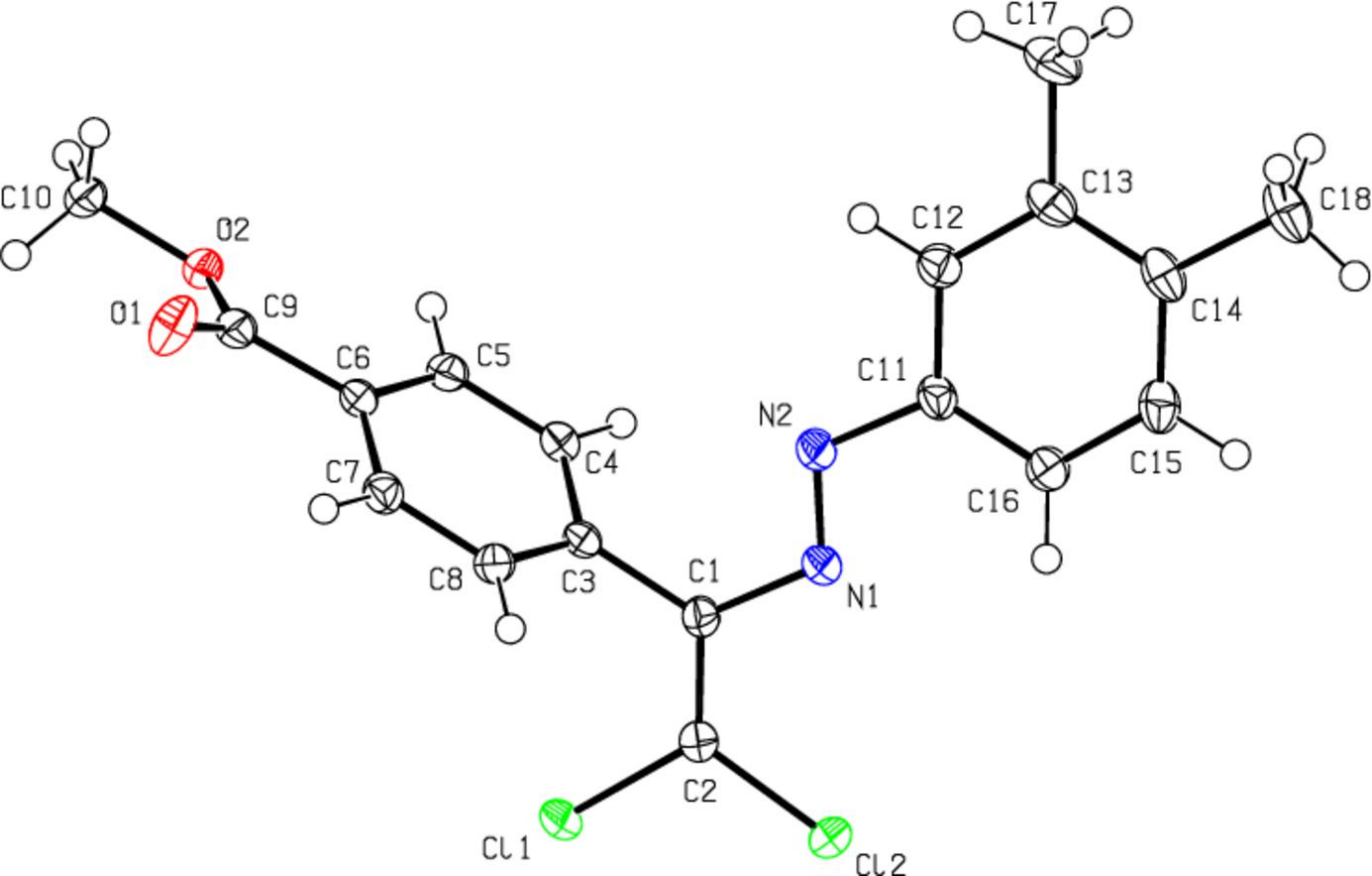
The mol­ecular structure of (**III**), showing the atom labelling and displacement ellipsoids drawn at the 50% probability level.

The central mol­ecular fragment of (**I**), C1/C2/N1/N2/C3/C11/Cl1/Cl2, is almost planar (Fig. 2 ▸), with a root-mean-square (r.m.s.) deviation of fitted atoms from the least-squares plane of 0.0471 Å. This plane forms dihedral angles of 23.39 (6) and 56.98 (4)°, respectively, with the planes of the phenyl (C11–C16) and methyl benzoate (C3–C8) rings. The central mol­ecular fragment of (**II**), C1/C2/N2/N1/C3/C11/Cl1/Cl2, is likewise planar with an r.m.s. deviation of fitted atoms of 0.0680 Å (Fig. 3 ▸) and makes dihedral angles of 14.87 (8) and 70.88 (3)°, respectively, with the planes of the 4-methyl­phenyl (C11–C16) and methyl benzoate (C3–C8) rings. The central mol­ecular fragment of (**III**), C1/C2/N1/N2/C3/C11/Cl1/Cl2 (r.m.s. deviation of fitted atoms = 0.0261 Å; Fig. 4 ▸) forms dihedral angles of 7.59 (6) and 69.58 (3)°, respectively, with the planes of the 3,4-di­methyl­phenyl (C11–C16) and methyl benzoate (C3–C8) rings.

All bond lengths and angles in (**I**), (**II**) and (**III**) are in agreement with those reported for the related azo compounds discussed in the *Database survey* section.

## 3. Supra­molecular features and Hirshfeld surface analysis

**Figure 5:**
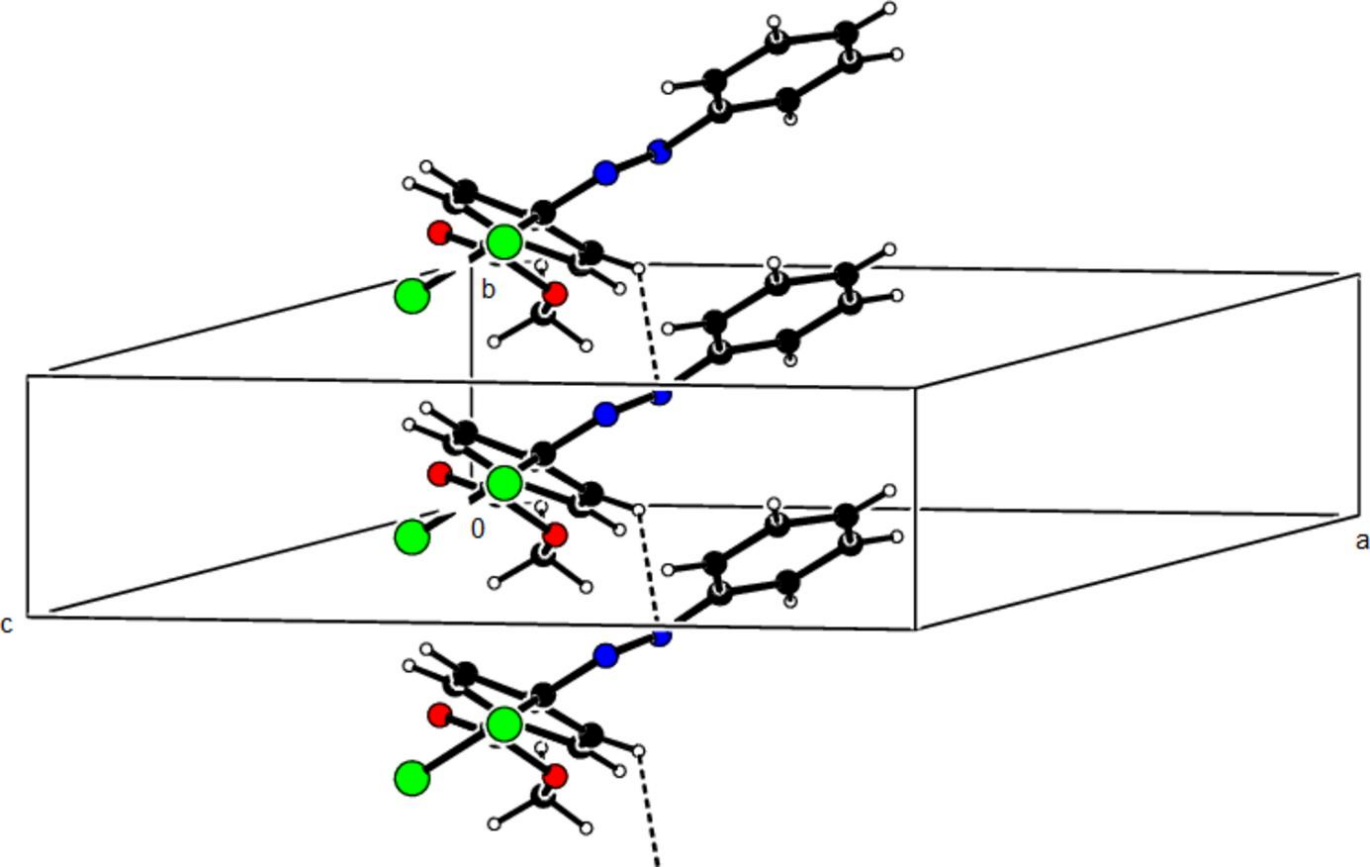
A general view of the C—H⋯N hydrogen bonds in the crystal structure of (**I**).

**Figure 6:**
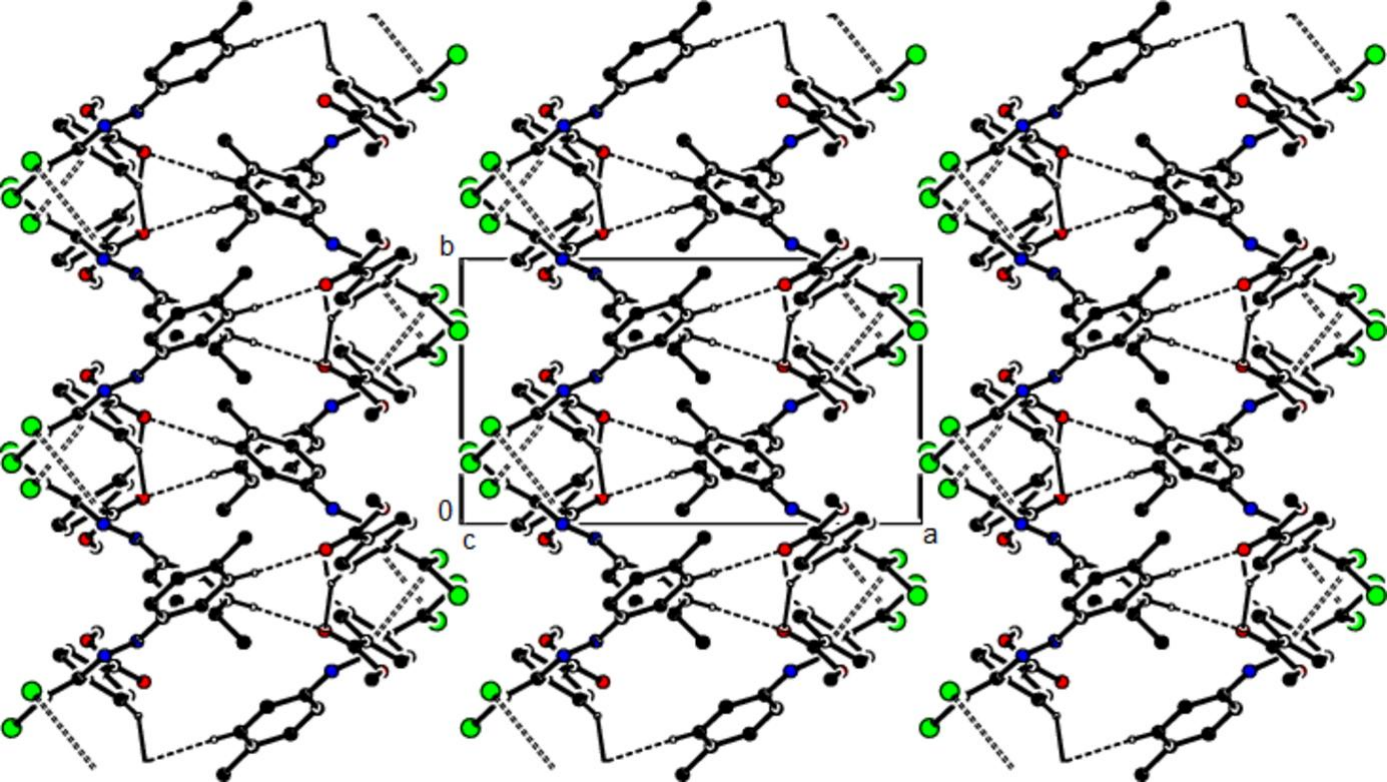
The crystal packing of (**II**) viewed along the *c* axis with inter­molecular C—H⋯O and C—Cl⋯π inter­actions shown as dashed lines.

**Figure 7:**
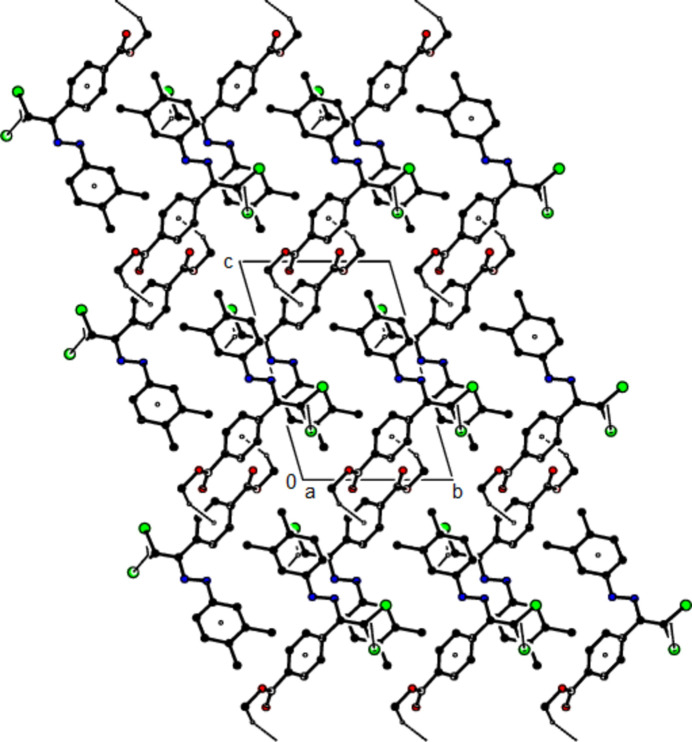
The crystal packing of (**III**) viewed along the *a* axis with C—H⋯π and C—Cl⋯π inter­actions shown as dashed lines.

In the crystal of (**I**), mol­ecules are linked by C—H⋯N hydrogen bonds, forming chains with *C*(6) motifs (Bernstein *et al.*, 1995 ▸) extending parallel to the *b* axis. Short inter­molecular Cl1⋯O1(−*x*, − 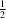 + *y*, 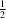 − *z*) contacts of 2.8421 (16) Å and weak van der Waals inter­actions between these chains stabilize the crystal structure (Table 1 ▸; Fig. 5 ▸). In the crystal of (**II**), mol­ecules are linked by C—H⋯O hydrogen bonds and C—Cl⋯π inter­actions [C2—Cl2⋯*Cg*1^*a*^: Cl2⋯*Cg*1^*a*^ = 3.5596 (8) Å, C2—Cl2⋯*Cg*1^*a*^ = 101.11 (6)°; symmetry code (*a*) *x*, 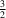 − *y*, − 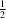 + *z*; *Cg*1 is the centroid of the C3–C8 benzene ring], forming layers parallel to (010) (Table 2 ▸; Fig. 6 ▸). Weak van der Waals inter­actions between these layers stabilize the mol­ecular packing. In the crystal of (**III**), mol­ecules are linked by C—H⋯π and C—Cl⋯π inter­actions, forming chains parallel to [011]. Furthermore, these chains are connected by C—Cl⋯π inter­actions [C2—Cl1⋯*Cg*2^*a*^: Cl1⋯*Cg*2^*a*^ = 3.5398 (8) Å, C2—Cl1⋯*Cg*2^*a*^ = 92.51 (5)°; C2—Cl2⋯*Cg*2^*b*^: Cl2⋯*Cg*2^*b*^ = 3.9545 (8) Å, C2—Cl2⋯*Cg*2^*b*^ = 88.18 (5)°; symmetry codes (*a*) −*x*, −*y*, 1 − *z*; (*b*) 1 − *x*, −*y*, 1 − *z*; *Cg*2 is the centroid of the 3,4-di­methyl­phenyl ring (C11–C16)] parallel to the *a* axis, forming layers parallel to (0 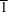 1) (Table 3 ▸; Figs. 7 ▸, 8 ▸ and 9 ▸). The stability of the mol­ecular packing is ensured by van der Waals forces between these layers.

**Figure 10:**
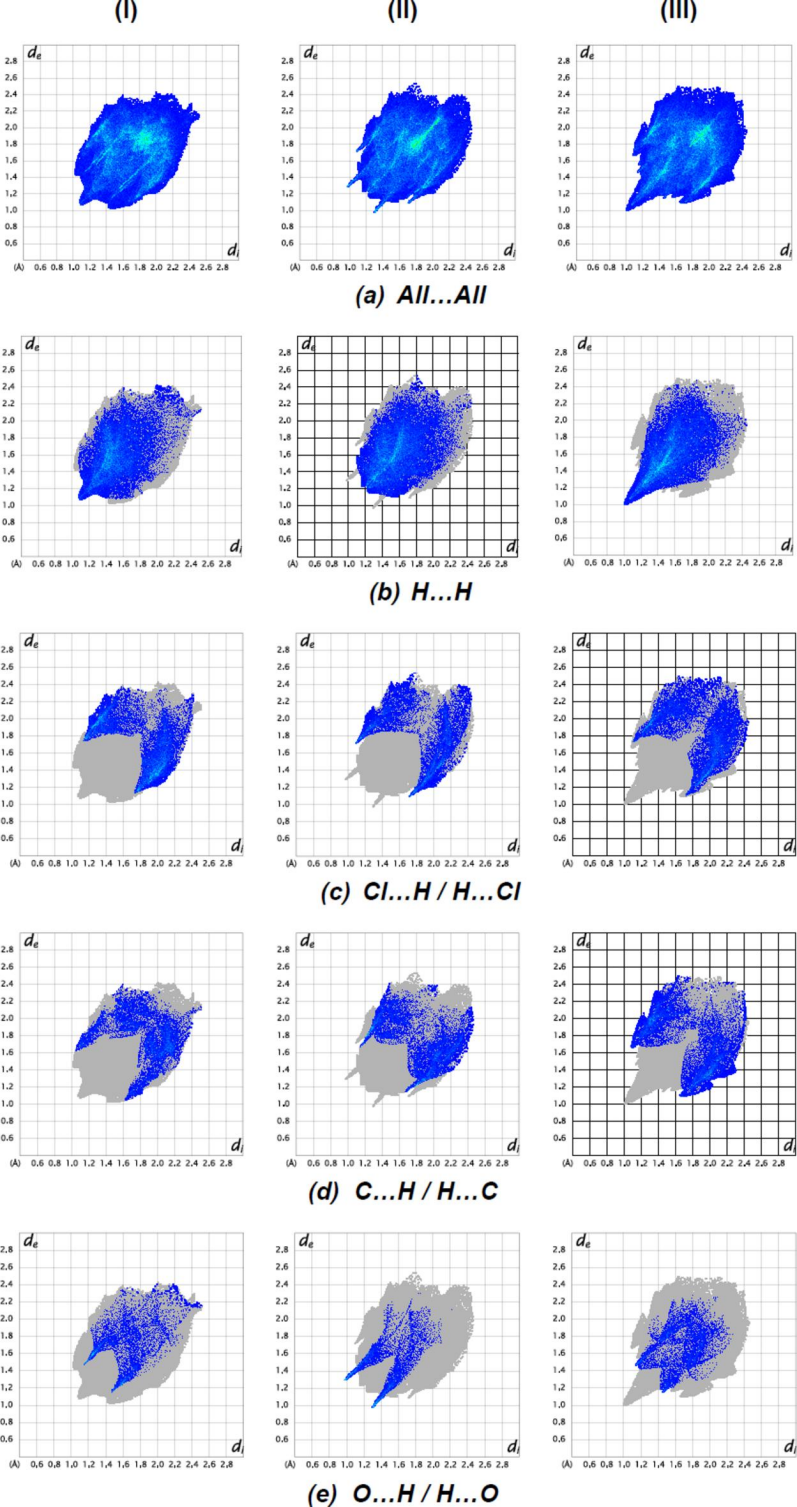
The full two-dimensional fingerprint plots for (**I**), (**II**) and (**III**), showing (*a*) all inter­actions, and delineated into (*b*) H⋯H, (*c*) Cl⋯H/H⋯Cl, (*d*) C⋯H/H⋯C, and (*e*) O⋯H/H⋯O inter­actions. The *d*_i_ and *d*_e_ values are the closest inter­nal and external distances (in Å) from given points on the Hirshfeld surface.

To qu­antify inter­molecular inter­actions between the mol­ecules in the crystal structures of (**I**), (**II**) and (**III**), Hirshfeld surface analyses were performed, together with two-dimensional fingerprint plots by using *CrystalExplorer* (Spackman *et al.*, 2021 ▸). The two-dimensional fingerprint plots are shown in Fig. 10 ▸. Comparative inter­actions calculated for each compound are given in Table 3 ▸. The dominant inter­actions in the crystal packing of the title compounds are H⋯H [(**I**): 33.5%, (**II**): 39.7% and (**III**): 37.0%], Cl⋯H/H⋯Cl [(**I**): 20.5%, (**II**): 14.4% and (**III**): 19.1%], C⋯H/H⋯C [(**I**): 14.3%, (**II**): 14.5% and (**III**):16.0%] and O⋯H/H⋯O [(**I**): 8.1%, (**II**): 6.6% and (**III**): 8.7%]. These inter­actions play a crucial role in the overall stabilization of the crystal packing. The presence of different functional groups in the compounds leads to some differences in the remaining weak inter­actions.

## 4. Database survey

A search of the Cambridge Structural Database (CSD, version 5.42, update of September 2021; Groom *et al.*, 2016 ▸) for the *(E)-1-(2,2-di­chloro-1-phenyl­ethen­yl)-2-phenyl­diazene* moiety resulted in 36 hits. Eighteen compounds are closely related to the title compound, *viz*. those with CSD refcodes NIKXEO (Maharramov *et al.*, 2023 ▸), NIKXIS (Maharramov *et al.*, 2023 ▸), NIKXOY (Maharramov *et al.*, 2023 ▸), NIKXUE (Maharramov *et al.*, 2023 ▸), TAZDIL (Atioğlu *et al.*, 2022*a* ▸), HEHKEO (Akkurt *et al.*, 2022 ▸), ECUDAL (Atioğlu *et al.*, 2022*b* ▸), PAXDOL (Çelikesir *et al.*, 2022 ▸), CANVUM (Shikhaliyev *et al.*, 2021*d* ▸), EBUCUD (Shikhaliyev *et al.*, 2021*a* ▸), GUPHIL (Özkaraca *et al.*, 2020*a* ▸), DULTAI (Özkaraca *et al.*, 2020*b* ▸), XIZREG (Atioğlu *et al.*, 2019 ▸), HODQAV (Shikhaliyev *et al.*, 2019*a* ▸), HONBUK (Akkurt *et al.*, 2019 ▸), HONBOE (Akkurt *et al.*, 2019 ▸), LEQXOX (Shikhaliyev *et al.*, 2018 ▸) and LEQXIR (Shikhaliyev *et al.*, 2018 ▸).

In the crystal structures of NIKXEO and NIKXIS, mol­ecules are linked by C—H⋯π and C—Cl⋯π inter­actions, forming layers parallel to ( 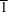 01), while mol­ecules of NIKXOY are linked by C—H⋯O contacts, C—H⋯π and C—Cl⋯π inter­actions, forming layers parallel to ( 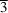 02). The stability of the mol­ecular packing is ensured by van der Waals forces between these layers. In the crystal structure of NIKXUE, mol­ecules are linked by C—H⋯π and C—Cl⋯π inter­actions, forming a tri-periodic network. The mol­ecules in TAZDIL are joined into layers parallel to (011) by C—H⋯O and C—H⋯F hydrogen bonds. C—Br⋯π and C—F⋯π contacts, as well as π—π stacking inter­actions strengthen the crystal packing. C—H⋯Br inter­actions connect the mol­ecules in the crystal of the polymorph-1 of HEHKEO, resulting in zigzag *C*(8) chains parallel to [100]. These chains are connected by C—Br⋯π inter­actions into layers parallel to (001). van der Waals inter­actions between the layers contribute to the crystal cohesion. In the crystals of ECUDAL, C—H⋯O hydrogen bonds link mol­ecules into chains. These chains are linked by face-to-face π–π stacking inter­actions, resulting in a layered structure. Short inter­molecular Br⋯O contacts and van der Waals inter­actions between the layers aid in the cohesion of the crystal packing. The mol­ecules in the crystal of PAXDOL are connected into chains running parallel to [001] by C—H⋯O hydrogen bonds. C—F⋯π contacts and π–π stacking inter­actions help to consolidate the crystal packing, and short Br⋯O [2.9828 (13) Å] distances are also observed. In CANVUM, the mol­ecules are linked by C—H⋯N inter­actions along [100], forming a *C*(6) chain. The mol­ecules are further connected by C—Cl⋯π inter­actions and face-to-face π–π stacking inter­actions, resulting in ribbons along [100]. The crystal structure of EBUCUD features short C—H⋯Cl and C—H⋯O contacts and C—H⋯π and van der Waals inter­actions. In GUPHIL, mol­ecules are associated into inversion dimers *via* short Cl⋯Cl contacts [3.3763 (9) Å]. In DULTAI, the crystal structure is stabilized by a short C—H⋯Cl contact, C—Cl⋯π and van der Waals inter­actions. In XIZREG, the mol­ecules are linked by C—H⋯O hydrogen bonds into zigzag chains running along [001]. The crystal packing also features C—Cl⋯π, C—F⋯π and N—O⋯π inter­actions. In HODQAV, mol­ecules are stacked in columns along [100] *via* weak C—H⋯Cl hydrogen bonds and face-to-face π—π stacking inter­actions. The crystal packing is further consolidated by short Cl⋯Cl contacts. In HONBUK and HONBOE, mol­ecules are linked through weak *X*⋯Cl contacts (*X* = Cl for HONBUK and Br for HONBOE), C—H⋯Cl and C—Cl⋯π inter­actions into sheets parallel to (001). Additional van der Waals inter­actions consolidate the three-dimensional packing. In the crystals of LEQXOX, C—H⋯N and short Cl⋯Cl contacts are observed and in LEQXIR, C—H⋯N and C—H⋯O hydrogen bonds and short C—Cl⋯O contacts occur.

## 5. Synthesis and crystallization

Dyes (**I**), (**II**) and (**III**) were synthesized according to a literature protocol (Maharramov *et al.*, 2018 ▸).

For dye (**I**), a 20 ml screw-neck vial was charged with DMSO (10 ml), methyl (*E*)-4-[(2-phenyl­hydrazineyl­idene)meth­yl]benzoate (254 mg, 1 mmol), tetra­methyl­ethylenedi­amine (TMEDA) (295 mg, 2.5 mmol), CuCl (2 mg, 0.02 mmol) and CCl_4_ (1 mmol). After 1–3 h (until TLC analysis showed complete consumption of the corresponding Schiff base), the reaction mixture was poured into a ∼0.01 *M* solution of HCl (100 ml, pH = 2–3), and extracted with di­chloro­methane (3 × ∼20 ml). The combined organic phase was washed with water (3× ∼50 ml), brine (30 ml), dried over anhydrous Na_2_SO_4_ and concentrated *in vacuo* using a rotary evaporator. The residue was purified by column chromatography on silica gel using appropriate mixtures of hexane and di­chloro­methane (*v*/*v*: 5/1–3/1–1/1). A red solid was obtained (yield 72%); m.p. 375 K. ^1^H NMR (300 MHz, chloro­form-*d*) δ 8.16–8.10 (*m*, 2H), 7.77 (*dd*, *J* = 6.8, 3.0 Hz, 2H), 7.48–7.43 (*m*, 3H), 7.29 (*d*, *J* = 8.3 Hz, 2H), 3.97 (*s*, 3H). ^13^C NMR (75 MHz, CDCl_3_) δ 169.2, 137.4, 132.0, 131.8, 130.1, 129.3, 129.1, 123.6, 123.2, 121.3, 120.0, 52.2.

For dye (**II**), the procedure was the same as that for (**I**) using methyl (*E*)-4-{[2-(*p*-tol­yl)hydrazineyl­idene]meth­yl}benzoate (268 mg, 1 mmol). A red solid was obtained (yield 78%); m.p. 399 K. ^1^H NMR (300 MHz, chloro­form-*d*) δ 8.13 (*d*, *J* = 8.3 Hz, 2H), 7.69 (*d*, *J* = 8.2 Hz, 2H), 7.32–7.22 (*m*, 4H), 3.95 (*s*, 3H), 2.40 (*s*, 3H). ^13^C NMR (75 MHz, CDCl_3_) δ 166.6, 151.5, 150.9, 142.6, 137.6, 134.8, 130.2, 129.8, 129.3, 123.3, 52.2, 21.6.

For dye (**III**), the procedure was the same as that for (**I**) using methyl (*E*)-4-{[2-(3,4-di­methyl­phen­yl)hydrazineyl­idene]meth­yl}benzoate (282 mg, 1 mmol). A red solid was obtained (yield 73%); m.p. 405 K. ^1^H NMR (300 MHz, chloro­form-*d*) δ 8.14 (*d*, *J* = 8.2 Hz, 2H), 7.60–7.52 (*m*, 2H), 7.29 (*d*, *J* = 8.1 Hz, 2H), 7.20 (*d*, *J* = 8.0 Hz, 1H), 3.96 (*s*, 3H), 2.31 (*s*, 6H). ^13^C NMR (75 MHz, CDCl_3_) δ 151.5, 151.2, 141.4, 137.7, 137.4, 134.5, 130.3, 130.2, 129.3, 124.6, 120.7, 52.2, 20.0.

Compounds (**I**), (**II**), and (**III**) were dissolved in di­chloro­methane and then left at room temperature for slow evaporation; red crystals of all compounds suitable for X-rays started to form after *ca* 2 d.

## 6. Refinement

Crystal data, data collection and structure refinement details are summarized in Table 5. In all three compounds (**I**), (**II**) and (**III**), all H atoms were positioned geometrically and treated as riding atoms, with C—H = 0.95–0.98 Å and *U*_iso_(H) = 1.2*U*_eq_(C) or 1.5*U*_eq_(C-meth­yl).

## Supplementary Material

Crystal structure: contains datablock(s) I, II, III, global. DOI: 10.1107/S2056989024000732/wm5708sup1.cif

Structure factors: contains datablock(s) I. DOI: 10.1107/S2056989024000732/wm5708Isup2.hkl

Structure factors: contains datablock(s) II. DOI: 10.1107/S2056989024000732/wm5708IIsup3.hkl

Structure factors: contains datablock(s) III. DOI: 10.1107/S2056989024000732/wm5708IIIsup4.hkl

Supporting information file. DOI: 10.1107/S2056989024000732/wm5708Isup5.cml

Supporting information file. DOI: 10.1107/S2056989024000732/wm5708IIsup6.cml

Supporting information file. DOI: 10.1107/S2056989024000732/wm5708IIIsup7.cml

CCDC references: 2327446, 2327447, 2327448

Additional supporting information:  crystallographic information; 3D view; checkCIF report

## Figures and Tables

**Figure 8:**
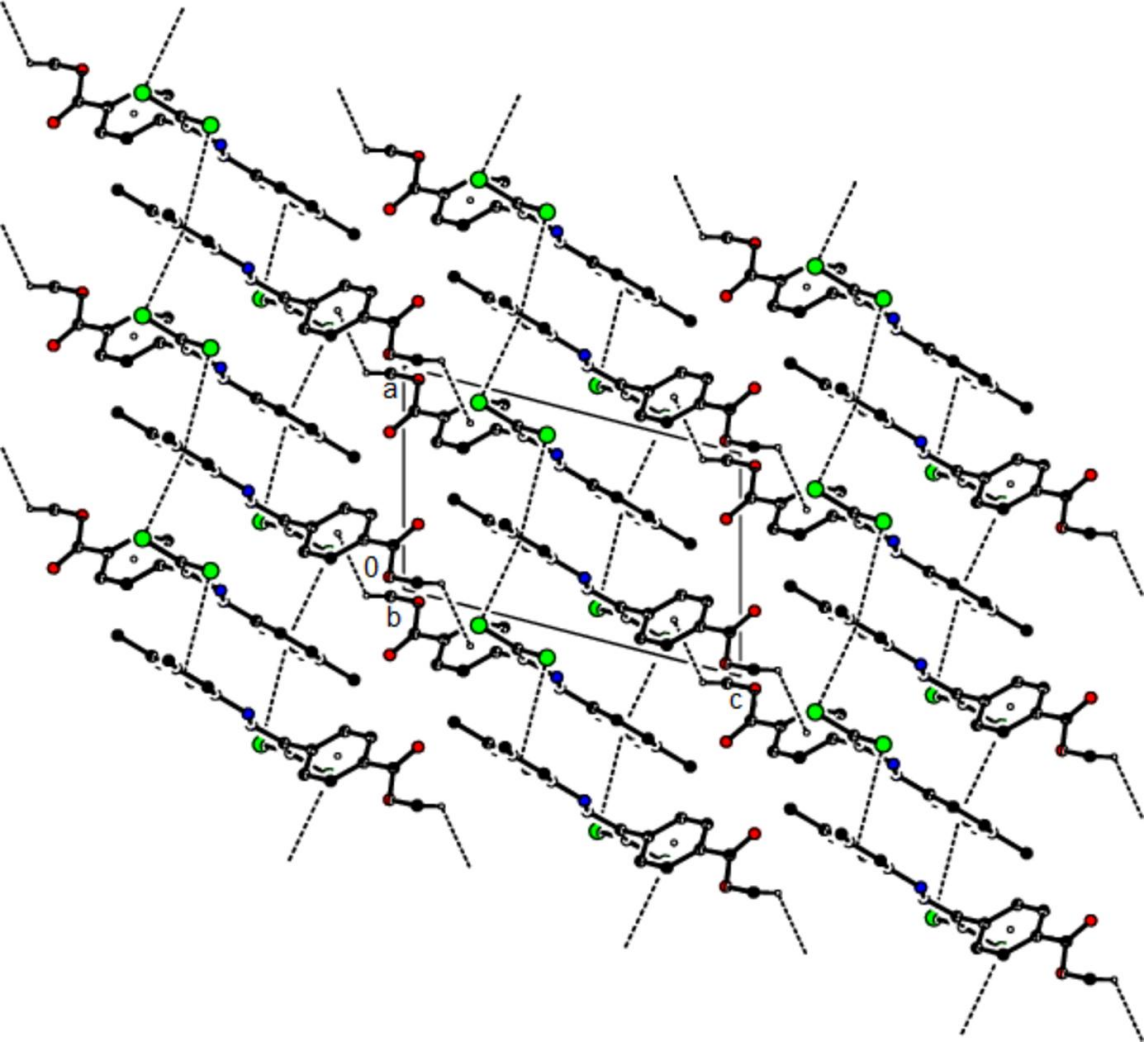
The crystal packing of (**III**) viewed along the *b* axis with C—H⋯π and C—Cl⋯π inter­actions shown as dashed lines.

**Figure 9:**
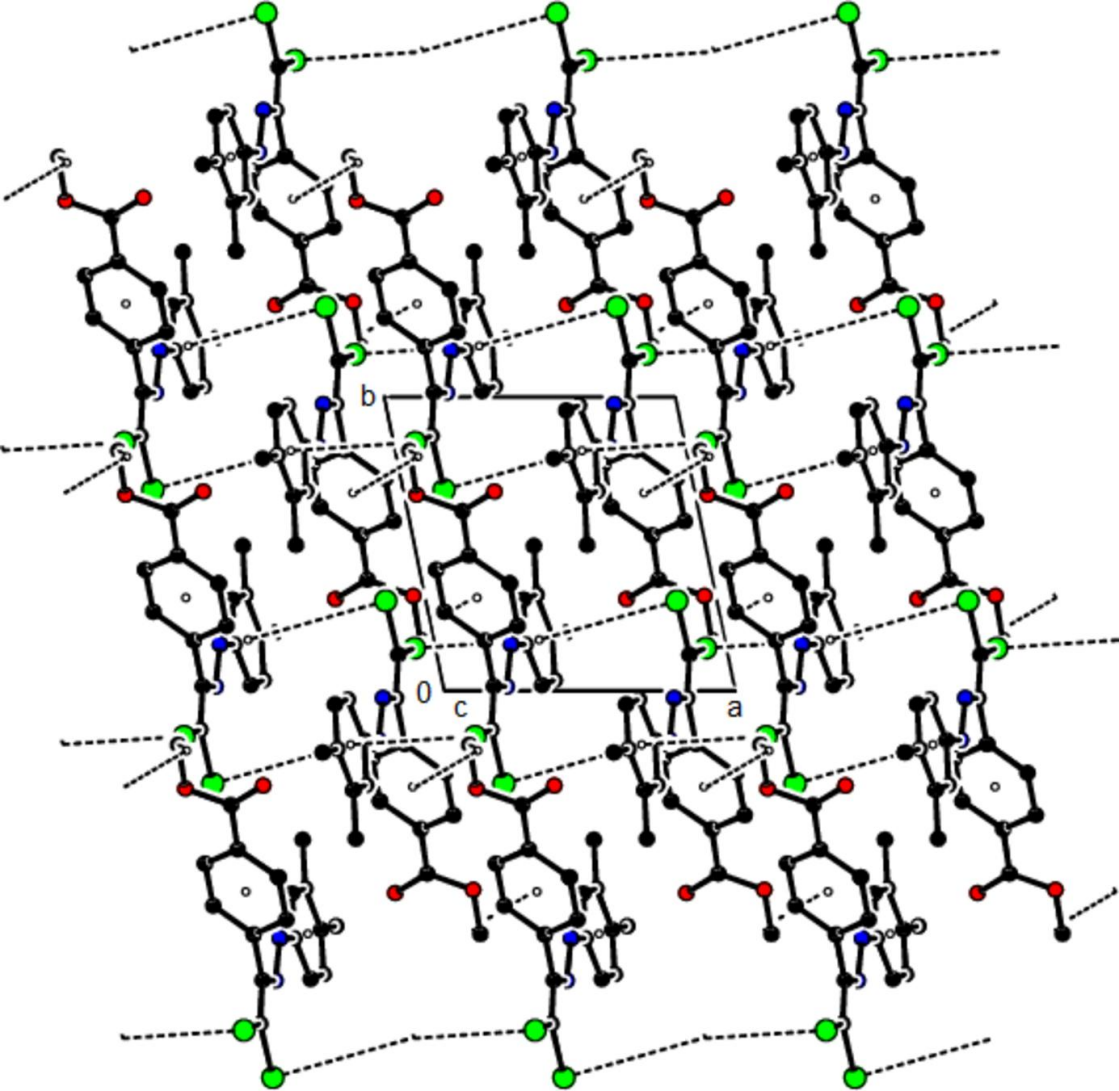
The crystal packing of (**III**) viewed along the *c* axis with C—H⋯π and C—Cl⋯π inter­actions.

**Table 1:** Hydrogen-bond geometry (Å, °) for (**I** [Chem scheme1])

*D*—H⋯*A*	*D*—H	H⋯*A*	*D*⋯*A*	*D*—H⋯*A*
C4—H4⋯N2^i^	0.95	2.54	3.191 (3)	126

Symmetry code: (i) 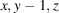 .

**Table 2:** Hydrogen-bond geometry (Å, °) for (**II** [Chem scheme1])

*D*—H⋯*A*	*D*—H	H⋯*A*	*D*⋯*A*	*D*—H⋯*A*
C4—H4⋯O1^i^	0.95	2.43	3.268 (2)	148
C13—H13⋯O1^ii^	0.95	2.40	3.309 (3)	159

Symmetry codes: (i) 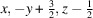 ; (ii) 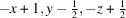 .

**Table 3:** Percentage contributions of inter­atomic contacts to the Hirshfeld surface for the title compounds

Contact		Percentage contribution	
	(**I**)	(**II**)	(**III**)
H⋯H	33.5	39.7	37.0
Cl⋯H/H⋯Cl	20.5	14.4	19.1
C⋯H/H⋯C	14.3	14.5	16.0
O⋯H/H⋯O	8.1	6.6	8.7
C⋯C	6.0	4.0	2.1
N⋯H/H⋯N	4.2	5.2	4.9
N⋯C/C⋯N	4.0	0.3	2.0
Cl⋯O/O⋯Cl	3.7	2.6	1.5
Cl⋯C/C⋯Cl	3.3	2.8	5.3
O⋯C/C⋯O	1.7	4.6	1.4
Cl⋯Cl	0.6	4.0	1.0
O⋯C/C⋯O	–	1.1	1.4
Cl⋯N/N.·Cl	–	0.8	0.4
O⋯C/C⋯O	–	–	0.2

**Table 4:** Experimental details

	(**I**)	(**II**)	(**III**)
Crystal data			
Chemical formula	C_16_H_12_Cl_2_N_2_O_2_	C_17_H_14_Cl_2_N_2_O_2_	C_18_H_16_Cl_2_N_2_O_2_
*M*_r_	335.18	349.20	363.23
Crystal system, space group	Monoclinic, *P*2_1_/*c*	Monoclinic, *P*2_1_/*c*	Triclinic, *P* 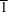
Temperature (K)	100	100	100
*a*, *b*, *c* (Å)	15.47572 (16), 4.16896 (4), 23.2257 (2)	15.6177 (2), 8.47502 (11), 13.10365 (17)	8.22057 (10), 8.53211 (9), 13.08729 (14)
α, β, γ (°)	90, 100.1964 (9), 90	90, 109.6555 (15), 90	103.9484 (9), 101.9047 (10), 98.0600 (9)
*V* (Å^3^)	1474.80 (2)	1633.34 (4)	854.09 (2)
*Z*	4	4	2
Radiation type	Cu *K*α	Cu *K*α	Cu *K*α
μ (mm^−1^)	4.04	3.67	3.53
Crystal size (mm)	0.22 × 0.13 × 0.11	0.26 × 0.19 × 0.17	0.20 × 0.15 × 0.09

Data collection			
Diffractometer	XtaLAB Synergy, Dualflex, HyPix	XtaLAB Synergy, Dualflex, HyPix	XtaLAB Synergy, Dualflex, HyPix
Absorption correction	Multi-scan (*CrysAlis PRO*; Rigaku OD, 2021 ▸)	Multi-scan (*CrysAlis PRO*; Rigaku OD, 2021 ▸)	Multi-scan (*CrysAlis PRO*; Rigaku OD, 2021 ▸)
*T*_min_, *T*_max_	0.404, 0.600	0.307, 0.530	0.349, 0.700
No. of measured, independent and observed [*I* > 2σ(*I*)] reflections	23940, 3151, 2823	28857, 3469, 3197	29808, 3632, 3340
*R*_int_	0.060	0.054	0.063
(sin θ/λ)_max_ (Å^−1^)	0.634	0.634	0.634

Refinement			
*R*[*F* ^2^ > 2σ(*F* ^2^)], *wR*(*F* ^2^), *S*	0.038, 0.103, 1.04	0.038, 0.108, 1.13	0.035, 0.097, 1.08
No. of reflections	3151	3469	3632
No. of parameters	200	211	220
H-atom treatment	H-atom parameters constrained	H-atom parameters constrained	H-atom parameters constrained
Δρ_max_, Δρ_min_ (e Å^−3^)	0.46, −0.32	0.30, −0.48	0.31, −0.46

Computer programs: *CrysAlis PRO* (Rigaku OD, 2021 ▸), *SHELXT* (Sheldrick, 2015*a* ▸), *SHELXL* (Sheldrick, 2015*b* ▸), *ORTEP-3 for Windows* (Farrugia, 2012 ▸) and *PLATON* (Spek, 2020 ▸).

## supplementary crystallographic information

### Methyl 4-{2,2-dichloro-1-[(E)-phenyldiazenyl]ethenyl}benzoate (I) Crystal data

**Table:**

C_16_H_12_Cl_2_N_2_O_2_	*F*(000) = 688
*M**_r_* = 335.18	*D*_x_ = 1.510 Mg m^−^^3^
Monoclinic, *P*2_1_/*c*	Cu *K*α radiation, λ = 1.54184 Å
*a* = 15.47572 (16) Å	Cell parameters from 12282 reflections
*b* = 4.16896 (4) Å	θ = 2.9–77.0°
*c* = 23.2257 (2) Å	µ = 4.04 mm^−^^1^
β = 100.1964 (9)°	*T* = 100 K
*V* = 1474.80 (2) Å^3^	Prism, red
*Z* = 4	0.22 × 0.13 × 0.11 mm

### Methyl 4-{2,2-dichloro-1-[(E)-phenyldiazenyl]ethenyl}benzoate (I) Data collection

**Table:**

XtaLAB Synergy, Dualflex, HyPix diffractometer	2823 reflections with *I* > 2σ(*I*)
Radiation source: micro-focus sealed X-ray tube	*R*_int_ = 0.060
φ and ω scans	θ_max_ = 77.8°, θ_min_ = 2.9°
Absorption correction: multi-scan (CrysAlisPro; Rigaku OD, 2021)	*h* = −19→19
*T*_min_ = 0.404, *T*_max_ = 0.600	*k* = −5→5
23940 measured reflections	*l* = −29→28
3151 independent reflections	

### Methyl 4-{2,2-dichloro-1-[(E)-phenyldiazenyl]ethenyl}benzoate (I) Refinement

**Table:**

Refinement on *F*^2^	0 restraints
Least-squares matrix: full	Hydrogen site location: inferred from neighbouring sites
*R*[*F*^2^ > 2σ(*F*^2^)] = 0.038	H-atom parameters constrained
*wR*(*F*^2^) = 0.103	*w* = 1/[σ^2^(*F*_o_^2^) + (0.0503*P*)^2^ + 1.3375*P*] where *P* = (*F*_o_^2^ + 2*F*_c_^2^)/3
*S* = 1.04	(Δ/σ)_max_ = 0.001
3151 reflections	Δρ_max_ = 0.46 e Å^−^^3^
200 parameters	Δρ_min_ = −0.32 e Å^−^^3^

### Methyl 4-{2,2-dichloro-1-[(E)-phenyldiazenyl]ethenyl}benzoate (I) Special details

**Table:**

Geometry. All esds (except the esd in the dihedral angle between two l.s. planes) are estimated using the full covariance matrix. The cell esds are taken into account individually in the estimation of esds in distances, angles and torsion angles; correlations between esds in cell parameters are only used when they are defined by crystal symmetry. An approximate (isotropic) treatment of cell esds is used for estimating esds involving l.s. planes.

### Methyl 4-{2,2-dichloro-1-[(E)-phenyldiazenyl]ethenyl}benzoate (I) Fractional atomic coordinates and isotropic or equivalent isotropic displacement parameters (Å^2^)

**Table:**

	*x*	*y*	*z*	*U*_iso_*/*U*_eq_	
Cl1	0.13479 (3)	0.05540 (11)	0.40331 (2)	0.02247 (13)	
Cl2	0.27874 (3)	0.32473 (12)	0.48326 (2)	0.02639 (14)	
O1	0.01936 (9)	0.1723 (4)	0.10978 (6)	0.0285 (3)	
O2	0.14455 (9)	−0.0786 (4)	0.10100 (6)	0.0268 (3)	
N1	0.34295 (10)	0.5658 (4)	0.38299 (7)	0.0216 (3)	
N2	0.38242 (10)	0.6368 (4)	0.34161 (7)	0.0211 (3)	
C1	0.26399 (11)	0.3911 (4)	0.36605 (8)	0.0207 (4)	
C2	0.22931 (12)	0.2761 (5)	0.41127 (8)	0.0214 (4)	
C3	0.22169 (11)	0.3354 (4)	0.30397 (8)	0.0197 (4)	
C4	0.26794 (11)	0.1764 (5)	0.26603 (8)	0.0210 (4)	
H4	0.327649	0.119411	0.279023	0.025*	
C5	0.22744 (12)	0.1012 (5)	0.20969 (8)	0.0220 (4)	
H5	0.259087	−0.011130	0.184514	0.026*	
C6	0.14025 (11)	0.1895 (4)	0.18962 (8)	0.0203 (4)	
C7	0.09485 (11)	0.3596 (4)	0.22649 (8)	0.0215 (4)	
H7	0.036219	0.427119	0.212652	0.026*	
C8	0.13490 (12)	0.4308 (5)	0.28326 (8)	0.0220 (4)	
H8	0.103337	0.544786	0.308278	0.026*	
C9	0.09401 (12)	0.0996 (5)	0.13000 (8)	0.0215 (4)	
C10	0.10264 (14)	−0.1883 (6)	0.04358 (8)	0.0293 (4)	
H10A	0.087522	−0.003127	0.017776	0.044*	
H10B	0.049121	−0.307201	0.046928	0.044*	
H10C	0.142947	−0.328838	0.027228	0.044*	
C11	0.46037 (11)	0.8199 (4)	0.36040 (8)	0.0209 (4)	
C12	0.51605 (12)	0.8486 (5)	0.31977 (8)	0.0228 (4)	
H12	0.500723	0.751252	0.282364	0.027*	
C13	0.59408 (13)	1.0194 (5)	0.33381 (9)	0.0255 (4)	
H13	0.632190	1.038116	0.306098	0.031*	
C14	0.61616 (12)	1.1624 (5)	0.38829 (9)	0.0258 (4)	
H14	0.670157	1.274595	0.398327	0.031*	
C15	0.55900 (13)	1.1414 (5)	0.42850 (8)	0.0264 (4)	
H15	0.573440	1.245282	0.465361	0.032*	
C16	0.48136 (12)	0.9698 (5)	0.41483 (8)	0.0233 (4)	
H16	0.442743	0.954405	0.442280	0.028*	

### Methyl 4-{2,2-dichloro-1-[(E)-phenyldiazenyl]ethenyl}benzoate (I) Atomic displacement parameters (Å^2^)

**Table:**

	*U*^11^	*U*^22^	*U*^33^	*U*^12^	*U*^13^	*U*^23^
Cl1	0.0167 (2)	0.0264 (2)	0.0244 (2)	−0.00357 (16)	0.00406 (15)	−0.00043 (16)
Cl2	0.0217 (2)	0.0355 (3)	0.0213 (2)	−0.00499 (17)	0.00171 (16)	−0.00103 (17)
O1	0.0183 (6)	0.0359 (8)	0.0291 (7)	0.0048 (6)	−0.0016 (5)	−0.0043 (6)
O2	0.0193 (6)	0.0382 (8)	0.0220 (6)	0.0047 (6)	0.0016 (5)	−0.0046 (6)
N1	0.0162 (7)	0.0232 (8)	0.0248 (7)	0.0001 (6)	0.0022 (6)	0.0017 (6)
N2	0.0153 (7)	0.0216 (7)	0.0260 (8)	0.0007 (6)	0.0025 (6)	0.0023 (6)
C1	0.0152 (8)	0.0208 (8)	0.0255 (9)	0.0019 (7)	0.0018 (7)	−0.0006 (7)
C2	0.0163 (8)	0.0233 (9)	0.0241 (9)	0.0006 (7)	0.0023 (6)	−0.0014 (7)
C3	0.0163 (8)	0.0202 (8)	0.0221 (9)	−0.0004 (7)	0.0023 (6)	0.0026 (7)
C4	0.0142 (8)	0.0244 (9)	0.0241 (9)	0.0035 (7)	0.0029 (6)	0.0038 (7)
C5	0.0166 (8)	0.0260 (9)	0.0240 (9)	0.0034 (7)	0.0049 (7)	0.0022 (7)
C6	0.0159 (8)	0.0220 (9)	0.0224 (8)	−0.0002 (7)	0.0021 (6)	0.0025 (7)
C7	0.0140 (8)	0.0221 (9)	0.0281 (9)	0.0019 (7)	0.0028 (7)	0.0016 (7)
C8	0.0153 (8)	0.0248 (9)	0.0261 (9)	0.0004 (7)	0.0044 (7)	−0.0011 (7)
C9	0.0175 (8)	0.0233 (9)	0.0237 (9)	0.0013 (7)	0.0038 (7)	0.0019 (7)
C10	0.0264 (10)	0.0391 (11)	0.0214 (9)	0.0002 (8)	0.0018 (7)	−0.0053 (8)
C11	0.0140 (8)	0.0219 (9)	0.0259 (9)	0.0017 (7)	0.0011 (6)	0.0034 (7)
C12	0.0205 (9)	0.0238 (9)	0.0246 (9)	0.0002 (7)	0.0053 (7)	0.0005 (7)
C13	0.0195 (9)	0.0274 (10)	0.0306 (10)	0.0009 (8)	0.0077 (7)	0.0020 (8)
C14	0.0155 (8)	0.0291 (10)	0.0319 (10)	−0.0030 (7)	0.0014 (7)	0.0046 (8)
C15	0.0233 (9)	0.0305 (10)	0.0241 (9)	−0.0045 (8)	0.0004 (7)	0.0017 (8)
C16	0.0189 (9)	0.0282 (9)	0.0227 (9)	−0.0010 (7)	0.0037 (7)	0.0035 (7)

### Methyl 4-{2,2-dichloro-1-[(E)-phenyldiazenyl]ethenyl}benzoate (I) Geometric parameters (Å, º)

**Table:**

Cl1—C2	1.7102 (19)	C7—C8	1.386 (3)
Cl2—C2	1.7231 (18)	C7—H7	0.9500
O1—C9	1.206 (2)	C8—H8	0.9500
O2—C9	1.343 (2)	C10—H10A	0.9800
O2—C10	1.450 (2)	C10—H10B	0.9800
N1—N2	1.262 (2)	C10—H10C	0.9800
N1—C1	1.417 (2)	C11—C12	1.391 (3)
N2—C11	1.429 (2)	C11—C16	1.396 (3)
C1—C2	1.349 (3)	C12—C13	1.390 (3)
C1—C3	1.492 (2)	C12—H12	0.9500
C3—C4	1.397 (3)	C13—C14	1.386 (3)
C3—C8	1.402 (2)	C13—H13	0.9500
C4—C5	1.383 (3)	C14—C15	1.398 (3)
C4—H4	0.9500	C14—H14	0.9500
C5—C6	1.397 (2)	C15—C16	1.386 (3)
C5—H5	0.9500	C15—H15	0.9500
C6—C7	1.394 (3)	C16—H16	0.9500
C6—C9	1.490 (3)		
			
C9—O2—C10	115.49 (15)	O1—C9—O2	123.12 (17)
N2—N1—C1	114.77 (15)	O1—C9—C6	124.62 (17)
N1—N2—C11	112.90 (15)	O2—C9—C6	112.26 (15)
C2—C1—N1	114.12 (16)	O2—C10—H10A	109.5
C2—C1—C3	121.99 (16)	O2—C10—H10B	109.5
N1—C1—C3	123.89 (16)	H10A—C10—H10B	109.5
C1—C2—Cl1	123.88 (14)	O2—C10—H10C	109.5
C1—C2—Cl2	122.96 (14)	H10A—C10—H10C	109.5
Cl1—C2—Cl2	113.11 (11)	H10B—C10—H10C	109.5
C4—C3—C8	119.01 (16)	C12—C11—C16	120.20 (17)
C4—C3—C1	119.81 (16)	C12—C11—N2	115.44 (16)
C8—C3—C1	121.15 (16)	C16—C11—N2	124.36 (16)
C5—C4—C3	120.48 (16)	C13—C12—C11	120.10 (18)
C5—C4—H4	119.8	C13—C12—H12	119.9
C3—C4—H4	119.8	C11—C12—H12	119.9
C4—C5—C6	120.35 (17)	C14—C13—C12	119.88 (18)
C4—C5—H5	119.8	C14—C13—H13	120.1
C6—C5—H5	119.8	C12—C13—H13	120.1
C7—C6—C5	119.42 (17)	C13—C14—C15	120.01 (18)
C7—C6—C9	119.19 (16)	C13—C14—H14	120.0
C5—C6—C9	121.38 (17)	C15—C14—H14	120.0
C8—C7—C6	120.28 (16)	C16—C15—C14	120.30 (18)
C8—C7—H7	119.9	C16—C15—H15	119.8
C6—C7—H7	119.9	C14—C15—H15	119.8
C7—C8—C3	120.38 (17)	C15—C16—C11	119.46 (17)
C7—C8—H8	119.8	C15—C16—H16	120.3
C3—C8—H8	119.8	C11—C16—H16	120.3
			
C1—N1—N2—C11	178.44 (15)	C4—C3—C8—C7	1.9 (3)
N2—N1—C1—C2	170.71 (16)	C1—C3—C8—C7	−176.03 (17)
N2—N1—C1—C3	−9.2 (3)	C10—O2—C9—O1	−1.8 (3)
N1—C1—C2—Cl1	−179.62 (13)	C10—O2—C9—C6	177.22 (16)
C3—C1—C2—Cl1	0.3 (3)	C7—C6—C9—O1	2.5 (3)
N1—C1—C2—Cl2	−2.2 (2)	C5—C6—C9—O1	−178.85 (19)
C3—C1—C2—Cl2	177.66 (14)	C7—C6—C9—O2	−176.46 (17)
C2—C1—C3—C4	−120.9 (2)	C5—C6—C9—O2	2.2 (3)
N1—C1—C3—C4	59.0 (3)	N1—N2—C11—C12	167.20 (16)
C2—C1—C3—C8	57.0 (3)	N1—N2—C11—C16	−14.1 (3)
N1—C1—C3—C8	−123.1 (2)	C16—C11—C12—C13	1.8 (3)
C8—C3—C4—C5	−2.9 (3)	N2—C11—C12—C13	−179.43 (17)
C1—C3—C4—C5	175.04 (17)	C11—C12—C13—C14	−0.3 (3)
C3—C4—C5—C6	1.4 (3)	C12—C13—C14—C15	−1.6 (3)
C4—C5—C6—C7	1.2 (3)	C13—C14—C15—C16	2.0 (3)
C4—C5—C6—C9	−177.42 (17)	C14—C15—C16—C11	−0.5 (3)
C5—C6—C7—C8	−2.2 (3)	C12—C11—C16—C15	−1.4 (3)
C9—C6—C7—C8	176.44 (17)	N2—C11—C16—C15	179.91 (18)
C6—C7—C8—C3	0.7 (3)		

### Methyl 4-{2,2-dichloro-1-[(E)-phenyldiazenyl]ethenyl}benzoate (I) Hydrogen-bond geometry (Å, º)

**Table:**

*D*—H···*A*	*D*—H	H···*A*	*D*···*A*	*D*—H···*A*
C4—H4···N2^i^	0.95	2.54	3.191 (3)	126
C5—H5···O2	0.95	2.40	2.726 (2)	100

Symmetry code: (i) *x*, *y*−1, *z*.

### Methyl 4-{2,2-dichloro-1-[(E)-(4-methylphenyl)diazenyl]ethenyl}benzoate (II) Crystal data

**Table:**

C_17_H_14_Cl_2_N_2_O_2_	*F*(000) = 720
*M**_r_* = 349.20	*D*_x_ = 1.420 Mg m^−^^3^
Monoclinic, *P*2_1_/*c*	Cu *K*α radiation, λ = 1.54184 Å
*a* = 15.6177 (2) Å	Cell parameters from 12932 reflections
*b* = 8.47502 (11) Å	θ = 3.0–77.0°
*c* = 13.10365 (17) Å	µ = 3.67 mm^−^^1^
β = 109.6555 (15)°	*T* = 100 K
*V* = 1633.34 (4) Å^3^	Prism, red
*Z* = 4	0.26 × 0.19 × 0.17 mm

### Methyl 4-{2,2-dichloro-1-[(E)-(4-methylphenyl)diazenyl]ethenyl}benzoate (II) Data collection

**Table:**

XtaLAB Synergy, Dualflex, HyPix diffractometer	3197 reflections with *I* > 2σ(*I*)
Radiation source: micro-focus sealed X-ray tube	*R*_int_ = 0.054
φ and ω scans	θ_max_ = 78.0°, θ_min_ = 3.0°
Absorption correction: multi-scan (CrysAlisPro; Rigaku OD, 2021)	*h* = −15→19
*T*_min_ = 0.307, *T*_max_ = 0.530	*k* = −10→10
28857 measured reflections	*l* = −16→16
3469 independent reflections	

### Methyl 4-{2,2-dichloro-1-[(E)-(4-methylphenyl)diazenyl]ethenyl}benzoate (II) Refinement

**Table:**

Refinement on *F*^2^	Hydrogen site location: inferred from neighbouring sites
Least-squares matrix: full	H-atom parameters constrained
*R*[*F*^2^ > 2σ(*F*^2^)] = 0.038	*w* = 1/[σ^2^(*F*_o_^2^) + (0.0579*P*)^2^ + 0.7484*P*] where *P* = (*F*_o_^2^ + 2*F*_c_^2^)/3
*wR*(*F*^2^) = 0.108	(Δ/σ)_max_ = 0.001
*S* = 1.13	Δρ_max_ = 0.30 e Å^−^^3^
3469 reflections	Δρ_min_ = −0.48 e Å^−^^3^
211 parameters	Extinction correction: SHELXL-2018/3 (Sheldrick, 2015b), Fc^*^=kFc[1+0.001xFc^2^λ^3^/sin(2θ)]^-1/4^
0 restraints	Extinction coefficient: 0.0012 (3)

### Methyl 4-{2,2-dichloro-1-[(E)-(4-methylphenyl)diazenyl]ethenyl}benzoate (II) Special details

**Table:**

Geometry. All esds (except the esd in the dihedral angle between two l.s. planes) are estimated using the full covariance matrix. The cell esds are taken into account individually in the estimation of esds in distances, angles and torsion angles; correlations between esds in cell parameters are only used when they are defined by crystal symmetry. An approximate (isotropic) treatment of cell esds is used for estimating esds involving l.s. planes.

### Methyl 4-{2,2-dichloro-1-[(E)-(4-methylphenyl)diazenyl]ethenyl}benzoate (II) Fractional atomic coordinates and isotropic or equivalent isotropic displacement parameters (Å^2^)

**Table:**

	*x*	*y*	*z*	*U*_iso_*/*U*_eq_	
Cl1	0.98647 (3)	0.77109 (5)	0.27683 (3)	0.02442 (14)	
Cl2	0.94376 (3)	0.63249 (5)	0.06421 (3)	0.02432 (14)	
O1	0.69644 (8)	0.59702 (16)	0.60175 (10)	0.0264 (3)	
O2	0.81960 (8)	0.44134 (15)	0.66154 (9)	0.0245 (3)	
N1	0.78451 (9)	0.49845 (18)	0.10125 (11)	0.0209 (3)	
N2	0.71406 (9)	0.44374 (18)	0.11566 (11)	0.0219 (3)	
C1	0.84094 (11)	0.5841 (2)	0.19136 (13)	0.0199 (3)	
C2	0.91421 (11)	0.6533 (2)	0.17879 (13)	0.0210 (3)	
C3	0.82373 (11)	0.5849 (2)	0.29668 (13)	0.0196 (3)	
C4	0.74921 (11)	0.6634 (2)	0.30912 (13)	0.0216 (3)	
H4	0.710519	0.725291	0.251576	0.026*	
C5	0.73197 (11)	0.6504 (2)	0.40622 (14)	0.0220 (3)	
H5	0.682177	0.705534	0.415595	0.026*	
C6	0.78742 (11)	0.5568 (2)	0.48977 (13)	0.0195 (3)	
C7	0.86273 (11)	0.4802 (2)	0.47817 (13)	0.0208 (3)	
H7	0.901389	0.418074	0.535632	0.025*	
C8	0.88054 (11)	0.4957 (2)	0.38166 (13)	0.0207 (3)	
H8	0.932079	0.444724	0.373610	0.025*	
C9	0.76178 (11)	0.5361 (2)	0.58862 (13)	0.0204 (3)	
C10	0.79809 (13)	0.4119 (2)	0.75872 (14)	0.0275 (4)	
H10A	0.839975	0.332619	0.802976	0.041*	
H10B	0.735518	0.373025	0.739045	0.041*	
H10C	0.804167	0.510044	0.800106	0.041*	
C11	0.65821 (11)	0.3536 (2)	0.02710 (13)	0.0215 (3)	
C12	0.57409 (12)	0.3086 (3)	0.03265 (15)	0.0296 (4)	
H12	0.556200	0.343233	0.091445	0.036*	
C13	0.51651 (13)	0.2138 (3)	−0.04704 (16)	0.0314 (4)	
H13	0.458899	0.185192	−0.043069	0.038*	
C14	0.54206 (12)	0.1597 (2)	−0.13325 (14)	0.0249 (4)	
C15	0.62625 (12)	0.2063 (2)	−0.13829 (14)	0.0233 (4)	
H15	0.644226	0.171215	−0.196914	0.028*	
C16	0.68413 (11)	0.3026 (2)	−0.05973 (13)	0.0217 (3)	
H16	0.741000	0.333796	−0.064785	0.026*	
C17	0.48059 (13)	0.0527 (3)	−0.21805 (16)	0.0337 (4)	
H17A	0.516980	−0.029287	−0.236871	0.050*	
H17B	0.448259	0.114318	−0.282843	0.050*	
H17C	0.436482	0.003205	−0.189705	0.050*	

### Methyl 4-{2,2-dichloro-1-[(E)-(4-methylphenyl)diazenyl]ethenyl}benzoate (II) Atomic displacement parameters (Å^2^)

**Table:**

	*U*^11^	*U*^22^	*U*^33^	*U*^12^	*U*^13^	*U*^23^
Cl1	0.0228 (2)	0.0257 (2)	0.0259 (2)	−0.00466 (15)	0.00965 (16)	−0.00107 (15)
Cl2	0.0258 (2)	0.0280 (2)	0.0246 (2)	0.00138 (15)	0.01564 (16)	0.00240 (15)
O1	0.0245 (6)	0.0358 (7)	0.0220 (6)	0.0048 (5)	0.0119 (5)	−0.0007 (5)
O2	0.0266 (6)	0.0303 (7)	0.0204 (6)	0.0048 (5)	0.0131 (5)	0.0057 (5)
N1	0.0212 (6)	0.0230 (7)	0.0204 (6)	−0.0009 (5)	0.0094 (5)	0.0002 (5)
N2	0.0223 (7)	0.0248 (7)	0.0202 (7)	−0.0011 (6)	0.0093 (5)	−0.0005 (6)
C1	0.0209 (7)	0.0203 (8)	0.0200 (8)	0.0022 (6)	0.0087 (6)	0.0015 (6)
C2	0.0219 (8)	0.0212 (8)	0.0214 (8)	0.0017 (6)	0.0092 (6)	0.0008 (6)
C3	0.0206 (7)	0.0205 (8)	0.0197 (8)	−0.0039 (6)	0.0095 (6)	−0.0019 (6)
C4	0.0213 (8)	0.0228 (8)	0.0209 (8)	0.0013 (6)	0.0075 (6)	0.0026 (6)
C5	0.0209 (8)	0.0231 (8)	0.0246 (8)	0.0008 (6)	0.0112 (6)	−0.0010 (6)
C6	0.0206 (7)	0.0205 (8)	0.0188 (7)	−0.0028 (6)	0.0086 (6)	−0.0019 (6)
C7	0.0199 (7)	0.0228 (8)	0.0206 (7)	−0.0001 (6)	0.0079 (6)	−0.0009 (6)
C8	0.0200 (7)	0.0221 (8)	0.0221 (8)	−0.0009 (6)	0.0099 (6)	−0.0030 (6)
C9	0.0199 (7)	0.0223 (8)	0.0201 (7)	−0.0026 (6)	0.0081 (6)	−0.0032 (6)
C10	0.0323 (9)	0.0340 (10)	0.0210 (8)	0.0027 (8)	0.0153 (7)	0.0043 (7)
C11	0.0227 (8)	0.0230 (8)	0.0196 (8)	−0.0004 (6)	0.0081 (6)	0.0001 (6)
C12	0.0265 (9)	0.0407 (11)	0.0261 (9)	−0.0058 (8)	0.0146 (7)	−0.0065 (8)
C13	0.0238 (8)	0.0438 (12)	0.0302 (9)	−0.0093 (8)	0.0138 (7)	−0.0067 (8)
C14	0.0260 (8)	0.0256 (9)	0.0226 (8)	−0.0018 (7)	0.0076 (7)	−0.0007 (7)
C15	0.0273 (8)	0.0244 (9)	0.0206 (8)	0.0019 (7)	0.0112 (7)	0.0008 (7)
C16	0.0222 (8)	0.0236 (8)	0.0214 (8)	0.0002 (6)	0.0101 (6)	0.0015 (7)
C17	0.0319 (9)	0.0391 (11)	0.0300 (9)	−0.0093 (8)	0.0105 (8)	−0.0090 (8)

### Methyl 4-{2,2-dichloro-1-[(E)-(4-methylphenyl)diazenyl]ethenyl}benzoate (II) Geometric parameters (Å, º)

**Table:**

Cl1—C2	1.7159 (17)	C7—H7	0.9500
Cl2—C2	1.7217 (16)	C8—H8	0.9500
O1—C9	1.207 (2)	C10—H10A	0.9800
O2—C9	1.339 (2)	C10—H10B	0.9800
O2—C10	1.4438 (19)	C10—H10C	0.9800
N1—N2	1.265 (2)	C11—C12	1.393 (2)
N1—C1	1.414 (2)	C11—C16	1.398 (2)
N2—C11	1.418 (2)	C12—C13	1.383 (3)
C1—C2	1.345 (2)	C12—H12	0.9500
C1—C3	1.492 (2)	C13—C14	1.396 (3)
C3—C8	1.391 (2)	C13—H13	0.9500
C3—C4	1.397 (2)	C14—C15	1.396 (2)
C4—C5	1.391 (2)	C14—C17	1.504 (2)
C4—H4	0.9500	C15—C16	1.385 (2)
C5—C6	1.393 (2)	C15—H15	0.9500
C5—H5	0.9500	C16—H16	0.9500
C6—C7	1.396 (2)	C17—H17A	0.9800
C6—C9	1.489 (2)	C17—H17B	0.9800
C7—C8	1.389 (2)	C17—H17C	0.9800
			
C9—O2—C10	115.59 (13)	O2—C10—H10A	109.5
N2—N1—C1	113.13 (13)	O2—C10—H10B	109.5
N1—N2—C11	113.70 (13)	H10A—C10—H10B	109.5
C2—C1—N1	116.03 (14)	O2—C10—H10C	109.5
C2—C1—C3	122.48 (15)	H10A—C10—H10C	109.5
N1—C1—C3	121.28 (14)	H10B—C10—H10C	109.5
C1—C2—Cl1	122.31 (13)	C12—C11—C16	119.63 (16)
C1—C2—Cl2	123.43 (13)	C12—C11—N2	115.85 (15)
Cl1—C2—Cl2	114.26 (9)	C16—C11—N2	124.45 (15)
C8—C3—C4	119.85 (15)	C13—C12—C11	120.30 (16)
C8—C3—C1	118.23 (14)	C13—C12—H12	119.8
C4—C3—C1	121.77 (15)	C11—C12—H12	119.8
C5—C4—C3	119.63 (15)	C12—C13—C14	120.76 (16)
C5—C4—H4	120.2	C12—C13—H13	119.6
C3—C4—H4	120.2	C14—C13—H13	119.6
C4—C5—C6	120.25 (15)	C15—C14—C13	118.40 (16)
C4—C5—H5	119.9	C15—C14—C17	120.88 (16)
C6—C5—H5	119.9	C13—C14—C17	120.72 (16)
C5—C6—C7	120.22 (14)	C16—C15—C14	121.44 (15)
C5—C6—C9	118.22 (14)	C16—C15—H15	119.3
C7—C6—C9	121.52 (15)	C14—C15—H15	119.3
C8—C7—C6	119.28 (15)	C15—C16—C11	119.45 (15)
C8—C7—H7	120.4	C15—C16—H16	120.3
C6—C7—H7	120.4	C11—C16—H16	120.3
C7—C8—C3	120.73 (15)	C14—C17—H17A	109.5
C7—C8—H8	119.6	C14—C17—H17B	109.5
C3—C8—H8	119.6	H17A—C17—H17B	109.5
O1—C9—O2	123.68 (15)	C14—C17—H17C	109.5
O1—C9—C6	124.15 (15)	H17A—C17—H17C	109.5
O2—C9—C6	112.17 (13)	H17B—C17—H17C	109.5
			
C1—N1—N2—C11	−178.24 (14)	C1—C3—C8—C7	−173.98 (15)
N2—N1—C1—C2	−176.18 (15)	C10—O2—C9—O1	1.4 (2)
N2—N1—C1—C3	8.9 (2)	C10—O2—C9—C6	−178.45 (14)
N1—C1—C2—Cl1	176.23 (12)	C5—C6—C9—O1	−1.0 (3)
C3—C1—C2—Cl1	−8.9 (2)	C7—C6—C9—O1	−178.47 (17)
N1—C1—C2—Cl2	−3.7 (2)	C5—C6—C9—O2	178.82 (14)
C3—C1—C2—Cl2	171.16 (13)	C7—C6—C9—O2	1.4 (2)
C2—C1—C3—C8	−69.6 (2)	N1—N2—C11—C12	−171.71 (16)
N1—C1—C3—C8	104.94 (19)	N1—N2—C11—C16	11.5 (2)
C2—C1—C3—C4	114.81 (19)	C16—C11—C12—C13	0.1 (3)
N1—C1—C3—C4	−70.6 (2)	N2—C11—C12—C13	−176.77 (18)
C8—C3—C4—C5	−0.6 (2)	C11—C12—C13—C14	1.0 (3)
C1—C3—C4—C5	174.91 (16)	C12—C13—C14—C15	−1.3 (3)
C3—C4—C5—C6	−1.5 (3)	C12—C13—C14—C17	178.15 (19)
C4—C5—C6—C7	2.4 (3)	C13—C14—C15—C16	0.6 (3)
C4—C5—C6—C9	−175.06 (15)	C17—C14—C15—C16	−178.87 (17)
C5—C6—C7—C8	−1.3 (2)	C14—C15—C16—C11	0.5 (3)
C9—C6—C7—C8	176.06 (15)	C12—C11—C16—C15	−0.9 (3)
C6—C7—C8—C3	−0.7 (3)	N2—C11—C16—C15	175.78 (16)
C4—C3—C8—C7	1.7 (3)		

### Methyl 4-{2,2-dichloro-1-[(E)-(4-methylphenyl)diazenyl]ethenyl}benzoate (II) Hydrogen-bond geometry (Å, º)

**Table:**

*D*—H···*A*	*D*—H	H···*A*	*D*···*A*	*D*—H···*A*
C4—H4···O1^i^	0.95	2.43	3.268 (2)	148
C13—H13···O1^ii^	0.95	2.40	3.309 (3)	159

Symmetry codes: (i) *x*, −*y*+3/2, *z*−1/2; (ii) −*x*+1, *y*−1/2, −*z*+1/2.

### Methyl 4-{2,2-dichloro-1-[(E)-(3,4-dimethylphenyl)diazenyl]ethenyl}benzoate (III) Crystal data

**Table:**

C_18_H_16_Cl_2_N_2_O_2_	*Z* = 2
*M**_r_* = 363.23	*F*(000) = 376
Triclinic, *P*1	*D*_x_ = 1.412 Mg m^−^^3^
*a* = 8.22057 (10) Å	Cu *K*α radiation, λ = 1.54184 Å
*b* = 8.53211 (9) Å	Cell parameters from 14210 reflections
*c* = 13.08729 (14) Å	θ = 3.6–77.0°
α = 103.9484 (9)°	µ = 3.53 mm^−^^1^
β = 101.9047 (10)°	*T* = 100 K
γ = 98.0600 (9)°	Prism, red
*V* = 854.09 (2) Å^3^	0.20 × 0.15 × 0.09 mm

### Methyl 4-{2,2-dichloro-1-[(E)-(3,4-dimethylphenyl)diazenyl]ethenyl}benzoate (III) Data collection

**Table:**

XtaLAB Synergy, Dualflex, HyPix diffractometer	3340 reflections with *I* > 2σ(*I*)
Radiation source: micro-focus sealed X-ray tube	*R*_int_ = 0.063
φ and ω scans	θ_max_ = 77.8°, θ_min_ = 3.6°
Absorption correction: multi-scan (CrysAlisPro; Rigaku OD, 2021)	*h* = −10→10
*T*_min_ = 0.349, *T*_max_ = 0.700	*k* = −8→10
29808 measured reflections	*l* = −16→16
3632 independent reflections	

### Methyl 4-{2,2-dichloro-1-[(E)-(3,4-dimethylphenyl)diazenyl]ethenyl}benzoate (III) Refinement

**Table:**

Refinement on *F*^2^	0 restraints
Least-squares matrix: full	Hydrogen site location: inferred from neighbouring sites
*R*[*F*^2^ > 2σ(*F*^2^)] = 0.035	H-atom parameters constrained
*wR*(*F*^2^) = 0.097	*w* = 1/[σ^2^(*F*_o_^2^) + (0.0537*P*)^2^ + 0.2636*P*] where *P* = (*F*_o_^2^ + 2*F*_c_^2^)/3
*S* = 1.08	(Δ/σ)_max_ = 0.001
3632 reflections	Δρ_max_ = 0.31 e Å^−^^3^
220 parameters	Δρ_min_ = −0.46 e Å^−^^3^

### Methyl 4-{2,2-dichloro-1-[(E)-(3,4-dimethylphenyl)diazenyl]ethenyl}benzoate (III) Special details

**Table:**

Geometry. All esds (except the esd in the dihedral angle between two l.s. planes) are estimated using the full covariance matrix. The cell esds are taken into account individually in the estimation of esds in distances, angles and torsion angles; correlations between esds in cell parameters are only used when they are defined by crystal symmetry. An approximate (isotropic) treatment of cell esds is used for estimating esds involving l.s. planes.

### Methyl 4-{2,2-dichloro-1-[(E)-(3,4-dimethylphenyl)diazenyl]ethenyl}benzoate (III) Fractional atomic coordinates and isotropic or equivalent isotropic displacement parameters (Å^2^)

**Table:**

	*x*	*y*	*z*	*U*_iso_*/*U*_eq_	
Cl1	0.07283 (5)	−0.14861 (4)	0.77715 (3)	0.02444 (11)	
Cl2	0.13915 (5)	−0.31047 (4)	0.57534 (3)	0.02637 (12)	
O1	0.30977 (14)	0.68544 (14)	1.04185 (9)	0.0315 (3)	
O2	0.04043 (13)	0.67433 (12)	0.95696 (8)	0.0208 (2)	
N1	0.21612 (15)	0.01951 (15)	0.54534 (10)	0.0198 (2)	
N2	0.26094 (15)	0.16265 (15)	0.53648 (10)	0.0208 (3)	
C1	0.17177 (17)	0.02110 (17)	0.64440 (11)	0.0189 (3)	
C2	0.13347 (18)	−0.12681 (18)	0.66254 (11)	0.0206 (3)	
C3	0.17331 (18)	0.17742 (17)	0.72590 (11)	0.0185 (3)	
C4	0.05481 (18)	0.27494 (17)	0.70373 (11)	0.0203 (3)	
H4	−0.026457	0.242988	0.635554	0.024*	
C5	0.05592 (18)	0.41897 (17)	0.78162 (12)	0.0203 (3)	
H5	−0.026072	0.484181	0.767260	0.024*	
C6	0.17767 (17)	0.46736 (17)	0.88079 (11)	0.0188 (3)	
C7	0.29835 (18)	0.37242 (17)	0.90121 (11)	0.0202 (3)	
H7	0.382930	0.406872	0.968032	0.024*	
C8	0.29570 (18)	0.22742 (17)	0.82428 (12)	0.0202 (3)	
H8	0.377651	0.162276	0.838900	0.024*	
C9	0.18624 (18)	0.61892 (17)	0.96841 (12)	0.0209 (3)	
C10	0.0464 (2)	0.82424 (18)	1.03931 (12)	0.0244 (3)	
H10A	−0.063270	0.858160	1.025427	0.037*	
H10B	0.135720	0.911518	1.037093	0.037*	
H10C	0.070772	0.804528	1.111150	0.037*	
C11	0.31099 (18)	0.16259 (18)	0.43840 (11)	0.0207 (3)	
C12	0.33518 (19)	0.31514 (18)	0.41637 (12)	0.0232 (3)	
H12	0.318547	0.409355	0.466000	0.028*	
C13	0.38330 (18)	0.3316 (2)	0.32285 (13)	0.0249 (3)	
C14	0.40929 (18)	0.1914 (2)	0.25099 (12)	0.0258 (3)	
C15	0.38626 (19)	0.0399 (2)	0.27461 (12)	0.0260 (3)	
H15	0.403729	−0.054527	0.225581	0.031*	
C16	0.33864 (19)	0.02371 (19)	0.36759 (12)	0.0231 (3)	
H16	0.325038	−0.079976	0.382923	0.028*	
C17	0.4088 (2)	0.4974 (2)	0.30023 (15)	0.0333 (4)	
H17A	0.376239	0.578206	0.355458	0.050*	
H17B	0.338263	0.488834	0.228149	0.050*	
H17C	0.528407	0.532926	0.302386	0.050*	
C18	0.4627 (2)	0.2029 (3)	0.14898 (14)	0.0363 (4)	
H18A	0.377011	0.242730	0.103353	0.055*	
H18B	0.473909	0.093688	0.108817	0.055*	
H18C	0.571996	0.279612	0.168542	0.055*	

### Methyl 4-{2,2-dichloro-1-[(E)-(3,4-dimethylphenyl)diazenyl]ethenyl}benzoate (III) Atomic displacement parameters (Å^2^)

**Table:**

	*U*^11^	*U*^22^	*U*^33^	*U*^12^	*U*^13^	*U*^23^
Cl1	0.0346 (2)	0.02094 (19)	0.02065 (18)	0.00298 (14)	0.01118 (14)	0.00900 (14)
Cl2	0.0402 (2)	0.01643 (18)	0.02316 (19)	0.00645 (14)	0.01064 (15)	0.00402 (13)
O1	0.0278 (6)	0.0277 (6)	0.0306 (6)	0.0074 (5)	0.0038 (5)	−0.0053 (5)
O2	0.0259 (5)	0.0182 (5)	0.0211 (5)	0.0070 (4)	0.0100 (4)	0.0059 (4)
N1	0.0231 (6)	0.0194 (6)	0.0185 (6)	0.0034 (5)	0.0077 (5)	0.0066 (5)
N2	0.0240 (6)	0.0199 (6)	0.0198 (6)	0.0025 (5)	0.0092 (5)	0.0060 (5)
C1	0.0214 (6)	0.0188 (7)	0.0171 (6)	0.0034 (5)	0.0071 (5)	0.0044 (5)
C2	0.0251 (7)	0.0191 (7)	0.0180 (6)	0.0041 (5)	0.0070 (5)	0.0049 (5)
C3	0.0234 (7)	0.0164 (6)	0.0187 (6)	0.0017 (5)	0.0108 (5)	0.0069 (5)
C4	0.0247 (7)	0.0203 (7)	0.0174 (6)	0.0040 (5)	0.0071 (5)	0.0070 (5)
C5	0.0242 (7)	0.0190 (7)	0.0216 (7)	0.0063 (5)	0.0097 (6)	0.0088 (5)
C6	0.0234 (7)	0.0170 (6)	0.0189 (7)	0.0029 (5)	0.0103 (5)	0.0067 (5)
C7	0.0216 (7)	0.0204 (7)	0.0186 (6)	0.0020 (5)	0.0063 (5)	0.0056 (5)
C8	0.0227 (7)	0.0191 (7)	0.0218 (7)	0.0060 (5)	0.0093 (5)	0.0070 (5)
C9	0.0248 (7)	0.0194 (7)	0.0219 (7)	0.0050 (5)	0.0105 (6)	0.0079 (6)
C10	0.0337 (8)	0.0179 (7)	0.0262 (7)	0.0080 (6)	0.0152 (6)	0.0066 (6)
C11	0.0212 (7)	0.0231 (7)	0.0190 (7)	0.0025 (5)	0.0085 (5)	0.0063 (6)
C12	0.0247 (7)	0.0226 (7)	0.0230 (7)	0.0022 (6)	0.0084 (6)	0.0069 (6)
C13	0.0214 (7)	0.0299 (8)	0.0252 (7)	0.0006 (6)	0.0064 (6)	0.0129 (6)
C14	0.0206 (7)	0.0376 (9)	0.0214 (7)	0.0024 (6)	0.0085 (6)	0.0114 (6)
C15	0.0245 (7)	0.0313 (8)	0.0235 (7)	0.0052 (6)	0.0119 (6)	0.0049 (6)
C16	0.0247 (7)	0.0226 (7)	0.0244 (7)	0.0042 (6)	0.0108 (6)	0.0072 (6)
C17	0.0366 (9)	0.0343 (9)	0.0335 (9)	0.0011 (7)	0.0109 (7)	0.0192 (7)
C18	0.0376 (9)	0.0506 (11)	0.0260 (8)	0.0047 (8)	0.0162 (7)	0.0157 (8)

### Methyl 4-{2,2-dichloro-1-[(E)-(3,4-dimethylphenyl)diazenyl]ethenyl}benzoate (III) Geometric parameters (Å, º)

**Table:**

Cl1—C2	1.7176 (14)	C10—H10A	0.9800
Cl2—C2	1.7148 (14)	C10—H10B	0.9800
O1—C9	1.2041 (19)	C10—H10C	0.9800
O2—C9	1.3422 (17)	C11—C16	1.396 (2)
O2—C10	1.4478 (17)	C11—C12	1.396 (2)
N1—N2	1.2651 (17)	C12—C13	1.393 (2)
N1—C1	1.4148 (17)	C12—H12	0.9500
N2—C11	1.4263 (17)	C13—C14	1.404 (2)
C1—C2	1.346 (2)	C13—C17	1.509 (2)
C1—C3	1.4901 (19)	C14—C15	1.397 (2)
C3—C8	1.390 (2)	C14—C18	1.509 (2)
C3—C4	1.397 (2)	C15—C16	1.384 (2)
C4—C5	1.392 (2)	C15—H15	0.9500
C4—H4	0.9500	C16—H16	0.9500
C5—C6	1.395 (2)	C17—H17A	0.9800
C5—H5	0.9500	C17—H17B	0.9800
C6—C7	1.390 (2)	C17—H17C	0.9800
C6—C9	1.4894 (19)	C18—H18A	0.9800
C7—C8	1.388 (2)	C18—H18B	0.9800
C7—H7	0.9500	C18—H18C	0.9800
C8—H8	0.9500		
			
C9—O2—C10	113.91 (11)	O2—C10—H10C	109.5
N2—N1—C1	112.85 (11)	H10A—C10—H10C	109.5
N1—N2—C11	113.33 (12)	H10B—C10—H10C	109.5
C2—C1—N1	116.08 (12)	C16—C11—C12	120.15 (13)
C2—C1—C3	121.77 (12)	C16—C11—N2	124.38 (13)
N1—C1—C3	122.12 (12)	C12—C11—N2	115.46 (13)
C1—C2—Cl2	124.19 (11)	C13—C12—C11	121.13 (14)
C1—C2—Cl1	122.44 (11)	C13—C12—H12	119.4
Cl2—C2—Cl1	113.37 (8)	C11—C12—H12	119.4
C8—C3—C4	119.86 (13)	C12—C13—C14	118.70 (14)
C8—C3—C1	119.43 (13)	C12—C13—C17	120.45 (15)
C4—C3—C1	120.70 (13)	C14—C13—C17	120.84 (14)
C5—C4—C3	119.96 (13)	C15—C14—C13	119.55 (13)
C5—C4—H4	120.0	C15—C14—C18	119.66 (15)
C3—C4—H4	120.0	C13—C14—C18	120.80 (15)
C4—C5—C6	119.88 (13)	C16—C15—C14	121.71 (14)
C4—C5—H5	120.1	C16—C15—H15	119.1
C6—C5—H5	120.1	C14—C15—H15	119.1
C7—C6—C5	119.93 (13)	C15—C16—C11	118.75 (14)
C7—C6—C9	117.03 (13)	C15—C16—H16	120.6
C5—C6—C9	123.04 (13)	C11—C16—H16	120.6
C8—C7—C6	120.22 (13)	C13—C17—H17A	109.5
C8—C7—H7	119.9	C13—C17—H17B	109.5
C6—C7—H7	119.9	H17A—C17—H17B	109.5
C7—C8—C3	120.10 (13)	C13—C17—H17C	109.5
C7—C8—H8	119.9	H17A—C17—H17C	109.5
C3—C8—H8	119.9	H17B—C17—H17C	109.5
O1—C9—O2	123.20 (13)	C14—C18—H18A	109.5
O1—C9—C6	124.17 (13)	C14—C18—H18B	109.5
O2—C9—C6	112.64 (12)	H18A—C18—H18B	109.5
O2—C10—H10A	109.5	C14—C18—H18C	109.5
O2—C10—H10B	109.5	H18A—C18—H18C	109.5
H10A—C10—H10B	109.5	H18B—C18—H18C	109.5
			
C1—N1—N2—C11	178.17 (11)	C10—O2—C9—O1	1.63 (19)
N2—N1—C1—C2	−175.79 (13)	C10—O2—C9—C6	−178.38 (11)
N2—N1—C1—C3	2.06 (19)	C7—C6—C9—O1	19.0 (2)
N1—C1—C2—Cl2	1.1 (2)	C5—C6—C9—O1	−161.01 (14)
C3—C1—C2—Cl2	−176.74 (11)	C7—C6—C9—O2	−161.04 (12)
N1—C1—C2—Cl1	−178.69 (10)	C5—C6—C9—O2	19.00 (18)
C3—C1—C2—Cl1	3.5 (2)	N1—N2—C11—C16	−11.1 (2)
C2—C1—C3—C8	67.37 (19)	N1—N2—C11—C12	170.08 (13)
N1—C1—C3—C8	−110.35 (15)	C16—C11—C12—C13	1.5 (2)
C2—C1—C3—C4	−113.54 (16)	N2—C11—C12—C13	−179.65 (13)
N1—C1—C3—C4	68.73 (18)	C11—C12—C13—C14	−0.7 (2)
C8—C3—C4—C5	−2.2 (2)	C11—C12—C13—C17	179.96 (14)
C1—C3—C4—C5	178.73 (12)	C12—C13—C14—C15	0.1 (2)
C3—C4—C5—C6	1.3 (2)	C17—C13—C14—C15	179.40 (14)
C4—C5—C6—C7	0.5 (2)	C12—C13—C14—C18	−179.55 (14)
C4—C5—C6—C9	−179.51 (12)	C17—C13—C14—C18	−0.2 (2)
C5—C6—C7—C8	−1.6 (2)	C13—C14—C15—C16	−0.2 (2)
C9—C6—C7—C8	178.48 (12)	C18—C14—C15—C16	179.48 (14)
C6—C7—C8—C3	0.7 (2)	C14—C15—C16—C11	0.9 (2)
C4—C3—C8—C7	1.2 (2)	C12—C11—C16—C15	−1.5 (2)
C1—C3—C8—C7	−179.74 (12)	N2—C11—C16—C15	179.72 (13)
